# Pathogen vacuole membrane contact sites – close encounters of the fifth kind

**DOI:** 10.1093/femsml/uqad018

**Published:** 2023-04-07

**Authors:** Simone Vormittag, Rachel J Ende, Isabelle Derré, Hubert Hilbi

**Affiliations:** Institute of Medical Microbiology, University of Zürich, Gloriastrasse 30, 8006 Zürich, Switzerland; Department of Microbiology, Immunology and Cancer Biology, University of Virginia, 1340 Jefferson Park Ave, Charlottesville, VA 22908, United States; Department of Microbiology, Immunology and Cancer Biology, University of Virginia, 1340 Jefferson Park Ave, Charlottesville, VA 22908, United States; Institute of Medical Microbiology, University of Zürich, Gloriastrasse 30, 8006 Zürich, Switzerland

**Keywords:** *Chlamydia*, *Coxiella*, *Dictyostelium discoideum*, endoplasmic reticulum, FFAT motif, *Legionella*, Legionnaires’ disease, lipid transfer proteins, membrane contact site, oxysterol binding proteins, pathogen vacuole, phosphoinositide, Sac1 phosphoinositide phosphatase, *Salmonella*, VAP

## Abstract

Vesicular trafficking and membrane fusion are well-characterized, versatile, and sophisticated means of ‘long range’ intracellular protein and lipid delivery. Membrane contact sites (MCS) have been studied in far less detail, but are crucial for ‘short range’ (10–30 nm) communication between organelles, as well as between pathogen vacuoles and organelles. MCS are specialized in the non-vesicular trafficking of small molecules such as calcium and lipids. Pivotal MCS components important for lipid transfer are the VAP receptor/tether protein, oxysterol binding proteins (OSBPs), the ceramide transport protein CERT, the phosphoinositide phosphatase Sac1, and the lipid phosphatidylinositol 4-phosphate (PtdIns(4)*P*). In this review, we discuss how these MCS components are subverted by bacterial pathogens and their secreted effector proteins to promote intracellular survival and replication.

## Teaser

Bacterial pathogens and their secreted effector proteins subvert membrane contact site components to promote intracellular survival and replication.

## Membrane contact sites in health and disease

**Figure 1.**
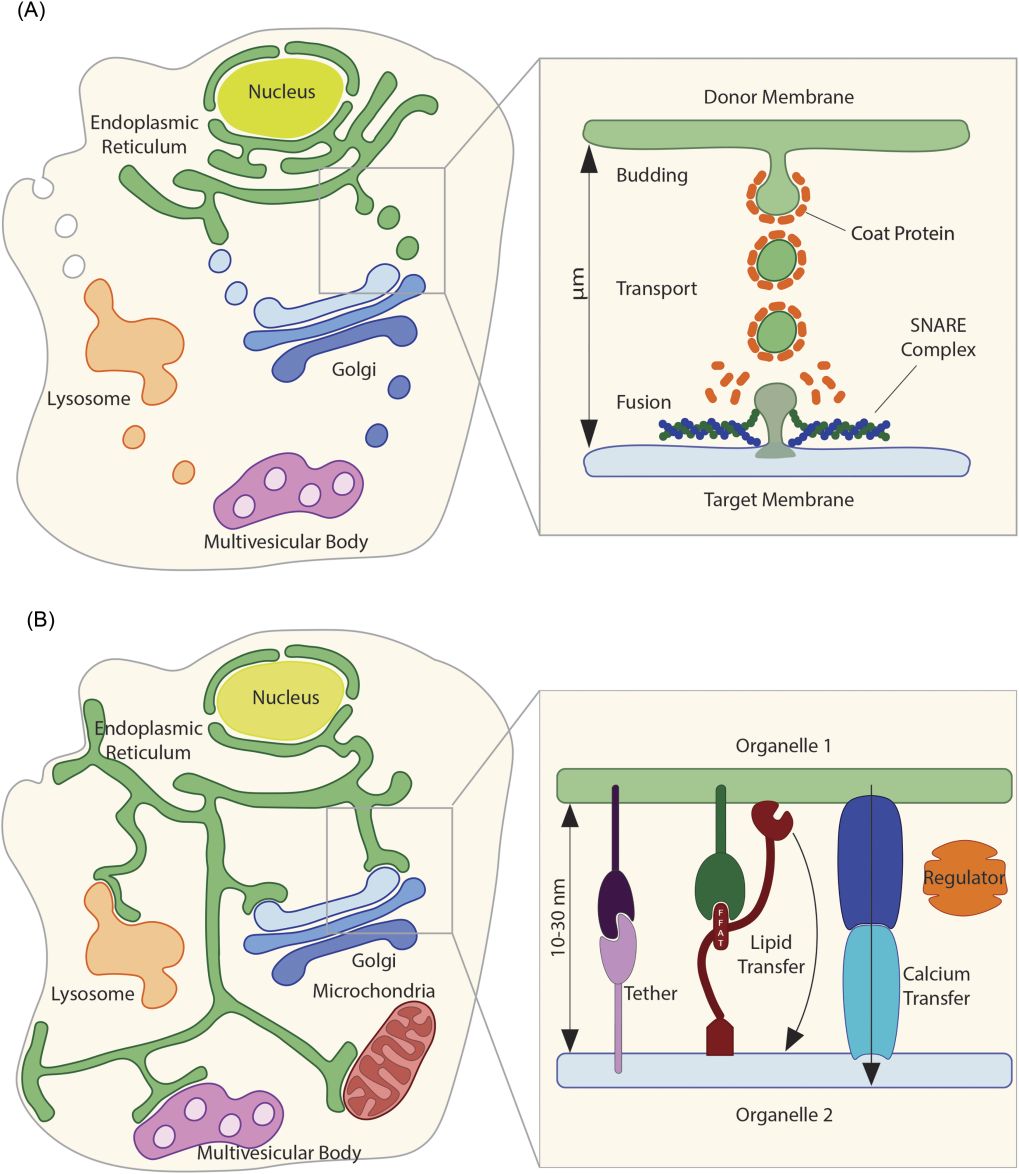
Vesicle trafficking and MCS formation. (**A**) Representation of the major vesicular trafficking events occurring between the ER, the Golgi, the plasma membrane, endosomes and multivesicular bodies. Inset: Representation of vesicle budding assisted by a coat protein from the donor membrane, long-range (µm scale) trafficking of the coated vesicle, and coat disassembly prior to SNARE-mediated fusion to the recipient membrane. (**B**) Representation of MCS occurring between the ER and the PM, the Golgi, mitochondria, endosomes and multivesicular bodies. Please note that additional MCS exist but are not represented here. Inset: Representation of the short-range (nm scale) transfer of small molecules at MCS, which is facilitated by structural (tether), functional (lipid/ion transfer) and regulatory components.

The ability for organelles to interact and communicate is essential for maintaining cellular homeostasis. One of the major means of intracellular communication, and the focus of research for many years, is vesicular trafficking. In general, vesicular trafficking involves two distinct membranes or membrane-bound organelles and occurs via three highly regulated steps: vesicle budding, transport, and fusion (Fig. 1A). During vesicle budding, coat proteins are recruited from the cytosol to the membrane surface and cause the deformation of the membrane to form a rounded bud (Springer et al. 1999, Kirchhausen 2000, Bonifacino and Lippincott-Schwartz 2003, McMahon and Mills 2004). After budding off from the membrane, vesicles are trafficked along cytoskeletal elements (actin and microtubules) by molecular motors such as dynein and kinesin to their target membrane (Hammer and Wu 2002, Matanis et al. 2002, Short et al. 2002). Upon arrival at the target membrane, the vesicle fuses with the target membrane through the presence of cognate SNARE (soluble N-ethylmaleimide-sensitive factor attachment protein receptors) proteins on the vesicle and target membrane (Jahn and Scheller 2006).

Another important means of inter-organelle communication is the direct interaction of two closely associated organelles, referred to as membrane contact sites (MCS) (Fig. 1B). Studies describing associations between organelles were first published in the 1950 s (Bernhard and Rouiller 1956, Copeland and Dalton 1959). However, a lack of functional significance for these associations at the time delayed further advances until the 1990 s, when associations between the ER and mitochondria were identified as sites of phospholipid synthesis and calcium transfer (Vance 1990, Rizzuto et al. 1998). MCS have since gained recognition due to their important implications in cell homeostasis, and several human diseases have been linked to MCS dysfunction (Area-Gomez et al. 2012, Stoica et al. 2014, Castro et al. 2018).

Specifically, MCS are zones of close apposition between the membranes of two organelles (10–30 nm) without membrane fusion (Prinz 2014, Scorrano et al. 2019). Functional inter-organellar contact is ubiquitous, with organelles forming MCS with at least one other organelle (Valm et al. 2017, Shai et al. 2018). MCS can be either homotypic, occurring between two identical organelles, or heterotypic, occurring between two different membranes or organelles. Similar contacts can also occur between membrane-bound organelles and non-membrane-bound organelles (Ma and Mayr 2018); however, these contacts are likely divergent from other cellular MCS and are not included in this review. The majority of cellular MCS include the ER, thus ER-containing MCS are the most well studied. For example, the ER forms membrane contact sites with mitochondria, endosomes, the Golgi, and the plasma membrane (PM) (Phillips and Voeltz 2016, Wu et al. 2018) (Fig. 1B). Membrane contacts sites not involving the ER have also been identified in recent years, such as mitochondria-PM, mitochondria-peroxisome, and lipid droplet (LD)-peroxisome MCS (Eisenberg-Bord et al. 2016).

The formation and function of MCS are dependent on the unique molecular composition of the two membranes involved. However, there are several general classes of MCS components: structural components, functional components, and the newly emerging class of regulatory components (Scorrano et al. 2019) (Fig. 1B). Structural components often act as tethers between the two membranes, maintaining them in close proximity (Scorrano et al. 2019). Functional components include lipid transfer proteins, ion channels, and metabolite channels/transporters, and these components have direct roles in ion, lipid, or metabolite exchange (Scorrano et al. 2019). Regulatory components act dynamically to regulate the formation of MCS and the activity of other MCS components (Honscher and Ungermann 2014, Honscher et al. 2014, Giorgi et al. 2015). Post-translational modification plays a major role in the regulation of protein interactions; thus, kinases and phosphatases often act as regulators at MCS (Kumagai et al. 2014, Duan and Walther 2015, Wu et al. 2018, Kors et al. 2022). It is important to note that these component classifications are not mutually exclusive, and many components can be placed in multiple classes.

MCS play diverse roles throughout the cell, some of the most notable being the regulation of intracellular calcium and non-vesicular lipid trafficking (Srikanth and Gwack 2012, Prakriya 2013, Hanada 2018, Wu et al. 2018). Although the bulk of lipid transfer likely occurs via vesicular trafficking, the non-vesicular lipid transfer occurring at ER-containing MCS plays an important role in maintaining lipid homeostasis in the absence of vesicular transport (Funato et al. 2020). MCS also play additional roles in organelle fission, such as the regulation of mitochondrial and endosomal fission at ER-mitochondria and ER-endosome MCS, respectively (Friedman et al. 2011, Murley et al. 2013, Rowland et al. 2014, Lewis et al. 2016). Additionally, MCS also have roles in organelle positioning (Friedman et al. 2010, Valm et al. 2017). For example, low levels of cellular cholesterol can result in endosomes forming MCS with the ER rather than continuing to be trafficked along microtubules (Rocha et al. 2009). Important for this review, there is increasing evidence that MCS play crucial roles in host pathogen interactions, with both viral (Amako et al. 2009, Roulin et al. 2014, McCune et al. 2017, Ishikawa-Sasaki et al. 2018) and bacterial pathogens (Auweter et al. 2012, Elwell and Engel 2012, Derré 2017, Justis et al. 2017, Stanhope and Derré 2018, Ende et al. 2022, Vormittag et al. 2023) using MCS to establish and maintain infection.

In this review we will introduce several MCS components, focusing on those most relevant to host pathogen interactions, as there are excellent reviews that cover additional MCS components (Scorrano et al. 2019, Prinz et al. 2020). We will also discuss how bacterial pathogens exploit these components and MCS formation to promote and support their intracellular survival.

## Components of membrane contact sites

### The VAP receptor

**Figure 2.**
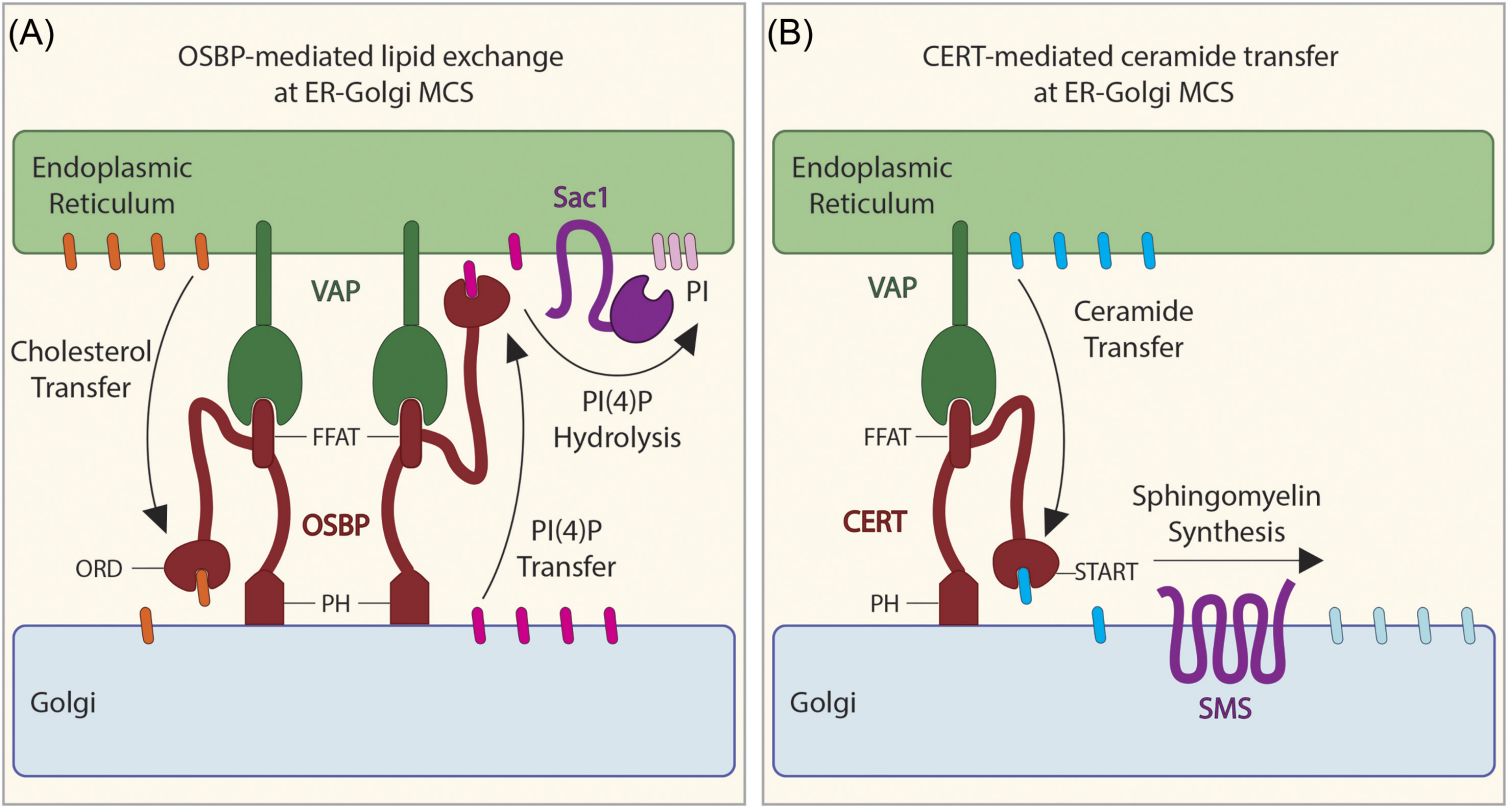
OSBP- and CERT-mediated lipid exchange at ER-Golgi MCS. (**A**) OSBP-meditated lipid exchange at ER-Golgi MCS implicates the FFAT motif and the ORD domain of OSBP, which binds to VAP and promotes lipid exchange, respectively. The Sac1 PI(4)*P* phosphatase maintains a PI(4)*P* lipid gradient between the two adjacent membranes. (**B**) CERT-mediated lipid exchange at ER-Golgi MCS implicates the FFAT motif and the START domain of CERT, which binds to VAP and promotes ceramide transfer, respectively. The sphingomyelin synthase SMS maintains a ceramide gradient between the two adjacent membranes.

VAP (Vesicle-associated membrane protein (VAMP)-associated) proteins are a family of ER-resident receptor/tether proteins that commonly play a role in the formation of ER-containing MCS through interaction with partner proteins on the opposing organelle (Murphy and Levine 2016) (Fig. 2). Two of the VAP family proteins, VAPA and VAPB, are highly homologous and are commonly referred to together as VAP (Murphy and Levine 2016). VAP proteins contain a globular domain with homology to major sperm protein (MSP domain), a predicted coiled-coil domain, and a transmembrane domain that anchors it in the ER (Kaiser et al. 2005). The formation of ER-MCS in the absence of VAP indicated that additional VAP variants or other proteins play a role in ER-MCS formation (Eden et al. 2016, Dong et al. 2016b). In fact, recent studies have identified three new homologs of VAP, motile sperm domain-containing proteins MOSPD1, MOSPD2, and MOSPD3, thus adding to the list of VAP-family proteins (Di Mattia et al. 2018, Cabukusta et al. 2020).

### FFAT motif containing protein partners of the VAP receptor

VAP family proteins form tethering complexes through the interaction of the MSP domain with FFAT motifs in partnering proteins such as oxysterol-binding protein (OSBP) (Loewen et al. 2003, Murphy and Levine 2016) (Fig. 2). FFAT (two phenylalanines (FF) in an Acidic Tract) motifs are linear peptide motifs with an E_1_-F_2_-F_3_-D_4_-A_5_-X_6_-E_7_ consensus core sequence flanked by adjacent acidic residues that create an acidic tract (Loewen et al. 2003, Loewen and Levine 2005). While deviation from the consensus core sequence is shown to be well tolerated, the residue in position two is considered essential and must be either a phenylalanine (F) or a tyrosine (Y) (Loewen and Levine 2005, Murphy and Levine 2016). Significant variation in the core FFAT motif sequence has become increasingly reported (Slee and Levine 2019, James and Kehlenbach 2021). Recent work identified phospho-FFAT motifs, where the residue in the fourth position of the motif core is a phosphorylatable serine or threonine, which upon phosphorylation favored the interaction with VAP (Di Mattia et al. 2020). Additionally, MOSPD1 and MOSPD3 favor interactions with proteins containing FFAT motifs referred to as FFNT (two phenylalanines (FF) in a Neutral Tract) motifs, where the residues flanking the core of the FFAT motif are neutral amino acids rather than acidic (Cabukusta et al. 2020).

The diversity of FFAT motif containing proteins contributes to the wide range of VAP-interacting partners, including soluble lipid transfer proteins as well as transmembrane proteins (James and Kehlenbach 2021). Thus, the role of VAP-FFAT interactions at MCS goes beyond tethering, especially during non-vesicular lipid transfer as discussed in the next section.

### Oxysterol binding and related proteins

In 1985 OSBP was identified as a receptor for oxysterols (Taylor and Kandutsch 1985) (Fig. 2A). Since then, a multitude of OSBP-related proteins (ORP) have been identified. OSBP and ORPs are conserved in mammalian cells, the yeast *Saccharomyces cerevisiae* (de Saint-Jean et al. 2011, Tong et al. 2013) and the social amoeba *Dictyostelium discoideum* (Fukuzawa and Williams 2002, Vormittag et al. 2023)—the evolutionary relationship among these proteins is outlined in Vormittag et al. 2023. Most of our knowledge about OSBP and ORPs structure and function comes from characterization of the mammalian proteins.

OSBP and the 11 human ORPs were classified into six subfamilies based on DNA sequence similarity and gene structure: family I (OSBP and ORP4), II (ORP1 and ORP2), III (ORP3, ORP6 and ORP7), IV (ORP5 and ORP8), V (ORP9) and VI (ORP10 and ORP11). All proteins contain multiple domains that are critical for membrane anchoring. With the exception of ORP5 and ORP8, which have transmembrane domains, most of the mammalian ORPs contain a pleckstrin homology (PH) domain (Fig. 2A) that interacts with phosphoinositide (PI) lipids and/or proteins in non-ER organelle membranes (Lemmon 2004, Olkkonen and Li 2013). Additionally, OSBP, ORP1-4, ORP6, ORP7, and ORP9 contain a FFAT motif (Fig. 2A), which is necessary for ER anchoring via binding to VAP (Wyles et al. 2002, Lehto et al. 2004, Wyles and Ridgway 2004, Lehto et al. 2005).

The primary known function of OSBP and ORPs is lipid transfer, which is mediated by core lipid binding domains (ORD) sharing 70% identify within each ORP family (Laitinen et al. 1999, Jaworski et al. 2001, Lehto et al. 2001), as well as the ORP signature motif EQVSHHPP. In addition to lipid binding, some ORD domains have been shown to interact with specific protein partners (Olkkonen et al. 2012, Pietrangelo and Ridgway 2019, D'Ambrosio et al. 2020). The ORD domains are also flanked by conserved regions of unknown functions. Several ORPs exist as long (L) and short variants (S), which differ in their cellular localization and interaction partners.

The long variant of ORP1, ORP1L, localizes to ER-late endosome (LE)/lysosome contact sites by interaction of its N-terminal ankyrin repeat domain (ARD) with the late endosome small GTPase Rab7 (Johansson et al. 2005). ORP1L also interacts with VAP through its FFAT motif after a conformational change due to low cholesterol conditions (Johansson et al. 2003, Johansson et al. 2005, Rocha et al. 2009, Vihervaara et al. 2011). The short variant of ORP1, ORP1S, lacks the PH domain as well as the FFAT motif and the ARD (Lehto et al. 2001, Loewen et al. 2003, Jansen et al. 2011). ORP1S localizes in the cytoplasm and in the nucleus and acts at ER-PM and LD-PM contact sites as well as at LE/lysosome-PM contact sites (Jansen et al. 2011, Zhao et al. 2020). Both ORP1 variants bind cholesterol or phosphatidylinositol 4-phosphate (PtdIns(4)*P*) through their ORD (Suchanek et al. 2007, Vihervaara et al. 2011, Zhao and Ridgway 2017, Dong et al. 2019).

ORP2 binds cholesterol, oxysterols, PtdIns(4)*P*, and PtdIns (4,5)*P*_2_ through its ORD (Wang et al. 2019). ORP2 only exists as a short variant consisting of an ORD and a FFAT motif (Lehto et al. 2001) and might act at ER-PM, LD-ER and endosome-PM contact sites (Laitinen et al. 2002, Hynynen et al. 2005, Hynynen et al. 2009, Wang et al. 2019).

ORP3 localizes to ER-PM contact sites after phosphorylation by protein kinase C (Lehto et al. 2008, Weber-Boyvat et al. 2015, Gulyas et al. 2020). ORP3 contains a FFAT motif, a PH domain, and an ORD that binds sterol, PtdIns(4)*P* and possibly phosphatidylcholine (PC) (Suchanek et al. 2007, D'Souza et al. 2020, Gulyas et al. 2020). Furthermore, ORP3 recruits the small GTPase R-Ras and thus contributes to the control of cell adhesion and migration (Lehto et al. 2008, Weber-Boyvat et al. 2015).

The long form of ORP4, ORP4L, localizes to ER-Golgi and ER-PM-contact sites (Zhong et al. 2016a, Zhong et al. 2016b, Pietrangelo and Ridgway 2018). The short variant, ORP4S, lacks the FFAT motif and interacts with vimentin intermediate filaments (Wang et al. 2002). An additional variant, ORPM, lacks a functional PH domain (Wyles et al. 2007, Charman et al. 2014). All ORP4 variants bind sterols or PtdIns(4)*P* through their ORD (Wyles et al. 2007, Goto et al. 2012, Charman et al. 2014).

ORP5 harbours a C-terminal transmembrane domain, thereby being constitutively anchored to the ER (Yan et al. 2008, Du et al. 2011). ORP5 acts at ER-PM contact sites (Maeda et al. 2013, Chung et al. 2015). OPR5 ORD binds phosphatidylserine (PS) and PtdIns(4)*P*, and its PH domain recognizes PtdIns(4)*P* as well as PtdIns(4,5)*P*_2_ (Ghai et al. 2017, Lee and Fairn 2018, Sohn et al. 2018). ORP5 also localizes to ER-mitochondria (Galmes et al. 2016), and ER-LD contact sites by interaction of its ORD with the LD monolayer (Du et al. 2020).

ORP6-11 have been studied in less detail. ORP6 localizes to ER-PM contact sites, its ORD likely binds PtdIns(4)*P* and it associates with ORP3 or itself (Lehto et al. 2004). Little is known about ORP7, except that it localizes to ER-PM contact sites (Lehto et al. 2004). ORP8 displays ER localization and acts at ER-mitochondria and ER-PM contact sites exchanging PS for PtdIns(4,5)*P*_2_ (Yan et al. 2008, Galmes et al. 2016, Ghai et al. 2017, Sohn et al. 2018). The long variant of ORP9, ORP9L, localizes to ER-Golgi contact sites and binds sterols and PtdIns(4)*P* through its ORD (Wyles and Ridgway 2004, Ngo and Ridgway 2009, Liu and Ridgway 2014). The short variant, ORP9S, lacks a PH domain and also localizes to ER-Golgi contact sites (Liu and Ridgway 2014). ORP10 acts at ER-Golgi contact sites, lacks a FFAT motif and binds PS as well as PtdIns(4)*P* through its ORD (Maeda et al. 2013, Venditti et al. 2019). ORP10 possibly heterodimerizes with ORP9 to overcome its inability to bind VAP (Nissila et al. 2012). ORP11 acts at ER-Golgi contact sites, lacks a FFAT motif and can interact with ORP9L. ORP11 ORD likely binds PtdIns(4)*P* and possibly sterols and PS (Suchanek et al. 2007, Maeda et al. 2013).

Unicellular eukaryotic fungi and protists, such as *S. cerevisiae* and *D. discoideum*, respectively, have also contributed to OSBP and ORPs characterization. In addition to elucidating their function at MCS, studies of the *S. cerevisiae* oxysterol-binding protein homolog (Osh) proteins were critical in providing structural insights into the mechanisms of lipid binding. The structure of the ORD domain of Osh4p revealed that it is comprised of a conserved β-barrel capped with a N-terminal lid (de Saint-Jean et al. 2011, Tong et al. 2013), in which cholesterol is bound in a ‘head-first’ orientation where the iso-octyl side chain interacts with the lid. In comparison, PtdIns(4)*P* is bound in a ‘tail-first’ orientation, where the inositol 4-phosphate headgroup interacts with two histidine residues close to the entrance (de Saint-Jean et al. 2011). *D. discoideum* produces short OSBPs, termed OSBP1-12, which contain ORD with the signature motif EQVSHHPP, but lack PH domains and FFAT motifs (Fukuzawa and Williams 2002, Vormittag et al. 2023).

Another important MCS lipid transport protein in mammalian cells is the ceramide transfer protein (CERT). CERT localizes to ER-Golgi contact sites (Hanada et al. 2003) (Fig. 2B). CERT interacts with VAP on the ER via its FFAT motif and with PtdIns(4)*P* at the Golgi through its PH domain. Once anchored at ER-Golgi MCS, the START domain of CERT binds, extracts, and transfers ceramide from the ER to the Golgi (Hanada et al. 2003, Kawano et al. 2006, Kudo et al. 2008). At the Golgi, a sphingomyelin synthase converts ceramide into sphingomyelin and thus maintains a ceramide gradient between the two adjacent membranes (Hanada 2018).

### The phosphoinositide phosphatase Sac1

Sac1 is an integral membrane protein, which anchors to the ER through two C-terminal transmembrane helices (Whitters et al. 1993, Nemoto et al. 2000, Konrad et al. 2002) (Fig. 2A). Sac1 is a phosphoinositide (PI) lipid phosphatase that contains the catalytical motif CX_5_R(T/S) (Nemoto et al. 2000). Sac1 binds the coat protein complex I and II (COPI and COPII, respectively), thereby cycling between the ER and the Golgi (Rohde et al. 2003, Weixel et al. 2005, Blagoveshchenskaya et al. 2008, Cheong et al. 2010). Human Sac1 preferentially dephosphorylates PtdIns(4)*P* and to a lesser extent also PtdIns(3)*P* (Rohde et al. 2003). Importantly, the hydrolysis of PtdIns(4)*P* results in a PI lipid concentration gradient between two membranes and is the driving force for the lipid exchange activity of OSBP (Mesmin et al. 2013) (Fig. 2A).

### Lipid transport at membrane contact sites

Various lipids, including sterols, ceramide, PS, and PIs are transported by lipid transfer proteins at MCS (Fig. 2). The precursor of PIs, phosphatidylinositol (PtdIns), is primarily synthesized in the ER and transported by vesicular trafficking or via lipid transfer proteins to distinct membranes (Di Paolo and De Camilli 2006). Phosphoinositide lipids contain a hydrophobic membrane anchor and a D-*myo*-inositol head group, which can be phosphorylated at position 3, 4, and/or 5 resulting in seven different PIs (De Matteis and Godi 2004, Behnia and Munro 2005, Raiborg et al. 2016). PI lipids can be modified by kinases, phosphatases, and lipases, which are recruited by small GTPases (Christoforidis et al. 1999, Godi et al. 1999, Jones et al. 2000, Murray et al. 2002).

The different PI lipids are spatially organized in the cell and show distinct subcellular localizations (Balla 2013). The PM contains PtdIns(4,5)*P*_2_, PtdIns(4)*P*, PtdIns(3)*P*, PtdIns(3,4)*P*_2_ and PtdIns(3,4,5)*P*_3_, while the Golgi is rich in PtdIns(4)*P* and to a lesser extent also harbours PtdIns(4,5)*P*_2_. Early endosomes are rich in PtdIns(3)*P*, late endosomes accumulate PtdIns(3,4)*P*_2_, and PtdIns(5)*P* is found on the nuclear membrane (De Matteis and Godi 2004, Behnia and Munro 2005, Di Paolo and De Camilli 2006, Weber et al. 2009b, Balla 2013). Sterols and PtdIns(4)*P* are synthesized at different membranes in the cell, giving rise to lipid gradients upon close contact of membranes of different lipid composition. Further in agreement with a counter exchange model of lipid transfer, reconstitution experiments with proteoliposomes showed that sterol and PtdIns(4)*P* are exchanged only when present on different membranes (*in trans*), but not on the same membrane (*in cis*) (de Saint-Jean et al. 2011, Mesmin et al. 2013, Moser von Filseck et al. 2015, Mesmin et al. 2017) (Fig. 2A).

Sterols comprise up to 40 mol% of lipids in the trans-Golgi and PM but only low levels (<5 mol%) in the ER (Radhakrishnan et al. 2008, van Meer et al. 2008, Mesmin and Maxfield 2009). This distribution arises in part from the presence of lipid components stabilizing cholesterol in the PM, like sphingolipids and phospholipids with saturated acyl chains, while the ER is rich in unsaturated lipids (van Meer et al. 2008, Holthuis and Menon 2014). Sterols increase the membrane thickness and decrease permeability to solutes. If present in excess, cholesterol is modified to cholesteryl ester by acyl coenzyme A and stored in the ER or LDs (Kreutzberger et al. 2015, Stratton et al. 2016).

Ceramide is synthesized in the ER and converted at the Golgi to sphingomyelin by the sphingomyelin synthase SMS (Fig. 2B), or to glycoceramides by various other enzymes (Jeckel et al. 1990, Wang et al. 2021). PS accumulates in the PM inner leaflets and in lower concentrations in the membranes of many other organelles (Leventis and Grinstein 2010). Upon undergoing apoptosis, cells flip PS to the outer leaflet, and the exposed PS is recognized by phagocytes (Brouckaert et al. 2004, Birge et al. 2016).

## Subversion of membrane contact sites by bacterial pathogens

A vast majority of intracellular bacterial pathogens create distinct replication-permissive compartments termed pathogen vacuoles. Although, the composition and features of these pathogen-derived vacuoles are specific to each pathogen, interactions with cellular membranes are a commonality. While the interception of host vesicular trafficking is a well-documented and characterized process, the formation of MCS, in particular with the ER, is emerging as a novel mechanism by which bacterial pathogens establish their replicative niche. In the following chapters, we review how the bacterial pathogens *Chlamydia, Legionella, Coxiella*, and possibly *Salmonella*, use secreted effector proteins to redirect cellular components of ER-Golgi, ER-PM, or ER-endosome contact sites to their distinct vacuoles, thereby assembling MCS between the pathogen vacuole and the ER.

### Membrane contact sites of the *Chlamydia* inclusion with the ER

**Figure 3.**
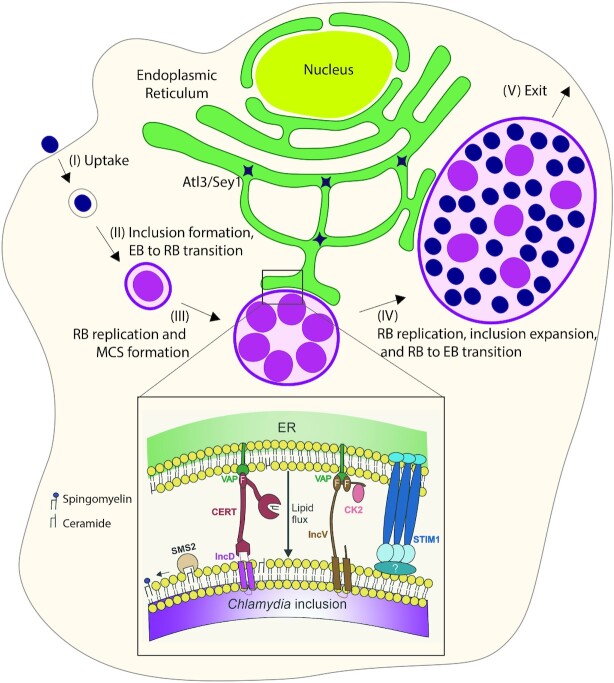
Formation of the *Chlamydia* ER-Inclusion MCS. Intracellular replication of *C. trachomatis* comprises the following steps: (I) uptake, (II) formation of the *Chlamydia* inclusion and transition of *Chlamydia* elementary bodies (EBs) to reticulate bodies (RBs), (III) RB replication and ER-Inclusion MCS formation, (IV) further RB replication, inclusion expansion and transition of RBs to EBs, and (V) bacterial exit through lytic or non-lytic (extrusion) pathways. The ER associates with the inclusion throughout the infection cycle. Several protein complexes localize to ER-Inclusion MCS. The T3SS-translocated *Chlamydia* Inc protein IncD recruits CERT to the inclusion membrane by binding to the PH domain of CERT. IncD bound CERT associates with the ER by binding to VAP via its FFAT motif. The IncD-CERT-VAP complex most likely functions in lipid transfer from the ER to the inclusion. The T3SS-translocated *Chlamydia* Inc protein IncV, which contains two FFAT motifs, directly associates with VAP. The IncV-VAP complex functions as a tether. The assembly of the IncV-VAP tether is positively regulated by IncV phosphorylation by the host kinase CK2. Through an unknown mechanism, the ER calcium sensor STIM1 also localizes to ER-Inclusion MCS.

The Gram-negative, obligate intracellular pathogen *Chlamydia trachomatis* is the causative agent of the most common sexually transmitted infection of bacterial origin in the United States and leading cause of infectious blindness worldwide (Wright et al. 2008, Mishori et al. 2012, Malhotra et al. 2013, Cheong et al. 2019). Upon entering the host cell, *Chlamydia* resides within a vacuole termed the inclusion (Moulder 1991, Abdelrahman and Belland 2005, Moore and Ouellette 2014, Gitsels et al. 2019) (Fig. 3). To establish and maintain its intracellular niche, *Chlamydia* interacts with host cell factors and organelles. These interactions, as well as maturation of the inclusion, are facilitated by *Chlamydia* type III secretion system (T3SS)-translocated effectors (Lara-Tejero and Galan 2019). A subset of these effectors, called inclusion membrane (Inc) proteins are embedded within the inclusion membrane and are characterized by the presence of two or more bi-lobed transmembrane domains and cytosolic tails that enable interactions with host factors (Bannantine et al. 2000, Dehoux et al. 2011, Lutter et al. 2012, Moore and Ouellette 2014, Bugalhao and Mota 2019).

Inc proteins have been shown to play a role in the direct interaction of the *Chlamydia* inclusion membrane with the ER (Fig. 3). This direct interaction was first observed by electron microscopy showing smooth and rough ER vesicles in close contact with the inclusion (Peterson and de la Maza 1988). Additional studies have identified patches of the ER maintained in close proximity (10-20 nm) to the inclusion in the absence of membrane fusion (Giles and Wyrick 2008, Derré et al. 2011, Elwell et al. 2011, Dumoux et al. 2012, Dumoux et al. 2015). Due to the morphological and molecular similarities with cellular MCS, these sites of direct contact have been referred to as ER-Inclusion MCS (Derré et al. 2011, Agaisse and Derré 2015).

Studies designed to characterize the molecular composition of ER-Inclusion MCS have identified several *Chlamydia* Inc proteins and host factors enriched at these sites. The Inc protein IncD interacts with the host lipid transfer protein CERT, which in turn binds VAP on the ER (Derré et al. 2011, Agaisse and Derré 2014) (Fig. 3). Depletion of CERT or VAP resulted in a significant decrease in inclusion size and infectious progeny production (Derré et al. 2011, Elwell et al. 2011). Based on the role of CERT and VAP at ER-Golgi MCS, the IncD-CERT-VAP complex is proposed to function in the non-vesicular trafficking of host lipids to the inclusion, a process essential for intracellular growth (Derré et al. 2011, Elwell et al. 2011, Agaisse and Derré 2014).

The host ER calcium sensor STIM1, a known component of ER-PM MCS, has also been shown to localize to ER-Inclusion MCS (Agaisse and Derré 2015). While its role at ER-Inclusion MCS remains unclear, STIM1 has been proposed to play a role in extrusion of the *Chlamydia* inclusion from the host cell (Nguyen et al. 2018). Recently STIM1 has also been implicated in preventing store-operated, calcium entry-dependent NFAT (nuclear factor of activated T cells) nuclear translocation in *C. trachomatis*-infected cells (Chamberlain et al. 2022). Finally, as this manuscript was under review, Cortina *et al*. reported the inclusion membrane protein IncS as STIM1-interacting partner at ER-Inclusion MCS (Cortina and Derré 2023), although the role of the IncS-STIM1 complex remains elusive.

The *Chlamydia* Inc protein IncV is also enriched at ER-Inclusion MCS (Stanhope et al. 2017) (Fig. 3). Work by Stanhope et al. demonstrated that IncV directly interacts with VAP through the presence of two FFAT motif cores in the C-terminal cytosolic tail of IncV (Stanhope et al. 2017). One of the FFAT motif cores is similar to the canonical sequence of eukaryotic FFAT motif cores, whereas the second motif diverges from the canonical sequence and was originally termed a non-canonical FFAT (Stanhope et al. 2017). Overexpression of IncV resulted in a dramatic increase in VAP and ER recruitment to the inclusion. Mutation of the essential position two of the IncV FFAT motifs disrupted the IncV-VAP interaction, supporting the notion that the IncV-VAP interaction functions as a tether between the inclusion and the ER (Stanhope et al. 2017). The IncV-VAP interaction serves as a prime example of molecular mimicry, where a bacterial pathogen displays eukaryotic motifs on the surface of its vacuole to allow for MCS formation.

Recently, Ende *et al*. showed that multiple layers of host cell kinase-mediated phosphorylation regulate the assembly of the IncV-VAP tethering complex and ER-Inclusion MCS formation (Ende et al. 2022). Previous work by Mirrashidi *et al*. predicted that IncV interacted with multiple host kinases, including all three subunits of the host kinase CK2 (Mirrashidi et al. 2015) (Fig. 3). Mutation of predicted CK2 phosphorylation motifs in the C-terminal region of IncV indicated that CK2 is recruited to the inclusion by IncV (Ende et al. 2022). Co-immunoprecipitation and electron microscopy further revealed that the phosphorylation of IncV by CK2 was required for establishing the IncV-VAP interaction at the inclusion (Ende et al. 2022). Phosphomimetic mutations in IncV indicated that phosphorylation of IncV by CK2 occurs within one of the FFAT motif cores and serine-rich tracts immediately upstream of IncV FFAT motif cores (Ende et al. 2022). Interestingly, IncV possesses phosphorylatable serine tracts, rather than acidic tracts, upstream of the two FFAT motif cores (Ende et al. 2022). Phosphomimetic mutation of these serine tracts to aspartic acid residues resulted in IncV remaining trapped within the bacteria and failing to be properly translocated, suggesting that the serine tracts allow for the mimicry of eukaryotic FFAT motifs while ensuring T3SS-mediated translocation of IncV to the inclusion membrane (Ende et al. 2022).

Overall, ER-Inclusion MCS resemble cellular MCS. The two membranes are tethered through VAP-FFAT interactions, and the presence of the IncD-CERT-VAP complex suggests that these MCS most likely play a role in non-vesicular lipid transfer. However, ER-Inclusion MCS do notably differ from cellular MCS. For example, in naïve cells, CERT and STIM1 localize to distinct MCS, namely ER-Golgi and ER-PM MCS, respectively. However, during *Chlamydia* infection these seemingly unrelated MCS components are both redirected to ER-Inclusion MCS, highlighting the capacity of the pathogen to bypass cellular ‘rules’. Additionally, unlike most eukaryotic FFAT motifs that contain tracts of acidic residues, the IncV FFAT motifs contain tracts of phosphorylatable serine residues, presumably to accommodate IncV secretion, further highlighting how pathogens have evolved to successfully hijack cellular molecules and pathways beneficial to their intracellular replication. Including these features in current FFAT motif identification algorithms could potentially identify additional FFAT motif containing proteins. Importantly, because pathogens often mimic cellular processes, the regulatory role of host kinase CK2 at ER-Inclusion MCS may indicate a role for CK2 at cellular MCS as well.

### The *Legionella*-containing vacuole-ER membrane contact sites

**Figure 4.**
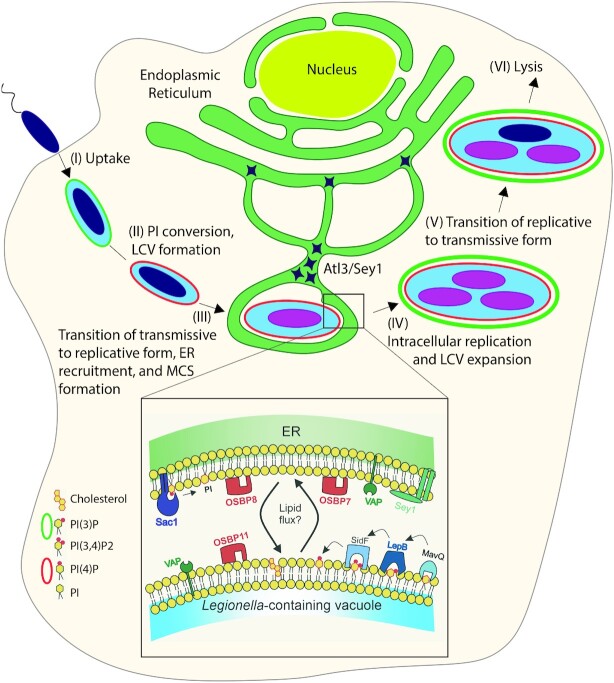
Formation of the *Legionella*-containing vacuole-ER MCS. Intracellular replication of *L. pneumophila* comprises the following steps: (I) uptake, (II) PI conversion and formation of the *Legionella*-containing vacuole (LCV), (III) transition of virulent/transmissive to replicative *L. pneumophila*, ER recruitment, and LCV-ER MCS formation, (IV) intracellular bacterial replication and LCV expansion, (V) transition of replicative to virulent/transmissive *L. pneumophila*, and (VI) host cell lysis and bacterial exit. At the LCV-ER MCS the VAP protein localizes to the LCV membrane as well as to the ER. OSBP7, OSBP8, the PI lipid phosphatase Sac1, and the large fusion GTPase Sey1 localize to the ER, while OSBP11 and T4BSS-translocated *L. pneumophila* effector proteins localize to the LCV.

*Legionella pneumophila* is a Gram-negative, rod-shaped, non-encapsulated, and flagellated bacterium, which upon inhalation of contaminated aerosols replicates in alveolar macrophages and can cause a severe pneumonia called Legionnaires’ disease (Newton et al. 2010, Hilbi et al. 2011, Mondino et al. 2020). *L. pneumophila* is a facultative intracellular bacterium that replicates in environmental free-living protozoa, such as *Acanthamoeba, Hartmannella, Vahlkampfia* and *Tetrahymena* species, as well as in the social amoeba *D. discoideum* (Steinert and Heuner 2005, Hoffmann et al. 2014a, Boamah et al. 2017, Swart et al. 2018). In mammalian and protozoan host cells, transmissive (virulent and motile) *L. pneumophila* establishes a unique compartment, the *Legionella*-containing vacuole (LCV), wherein which the bacteria switch to a replicative form (Isberg et al. 2009, Hubber and Roy 2010, Hilbi and Buchrieser 2022) (Fig. 4). As replication ceases, the bacteria switch back to the virulent form, and a transmissive bacterial subpopulation escapes the LCV and lyses the host cell (Striednig et al. 2021).

LCV formation is controlled by the bacterial Icm/Dot type IVB secretion system (T4BSS) (Segal et al. 2005, Kubori and Nagai 2016). The Icm/Dot T4BSS translocates approximately 330 different ‘effector’ proteins into host cells, where they subvert pivotal processes, including the endocytic, secretory, retrograde and autophagy pathways, cytoskeleton dynamics, metabolism, transcription, translation, and apoptosis (Ge and Shao 2011, Hilbi and Haas 2012, Finsel and Hilbi 2015, Escoll et al. 2016, Personnic et al. 2016, Bärlocher et al. 2017, Qiu and Luo 2017, Swart et al. 2020). A decisive step during LCV maturation is the diversion from the endocytic pathway and the interception of the secretory pathway, along with a PI lipid conversion from endosomal PtdIns(3)*P* to secretory PtdIns(4)*P* (Weber et al. 2006, Weber et al. 2014, Steiner et al. 2018, Swart and Hilbi 2020) (Fig. 4).

PI lipid conversion of the LCV is catalysed by several Icm/Dot-translocated effectors: the PtdIns 3-kinase MavQ (Li et al. 2021), the PtdIns(3)*P* 4-kinase LepB (Dong et al. 2016a), and the PtdIns(3,4)*P*_2_ 3-phosphatase SidF (Hsu et al. 2012), as well as possibly by host PI-metabolizing enzymes: the PtdIns 4-kinases PI4KIIIβ (Brombacher et al. 2009) and PI4KIIIα (Hubber et al. 2014), and the PtdIns(4,5)*P* 5-phosphatase OCRL (Weber et al. 2009a, Choi et al. 2021). A number of Icm/Dot-translocated effectors also anchor to the LCV membrane by binding to distinct PI lipids (Swart and Hilbi 2020). Accordingly, the ubiquitin ligase SidC (Weber et al. 2006, Ragaz et al. 2008, Dolinsky et al. 2014, Luo et al. 2015), the Rab1 GEF/AMPylase SidM (Brombacher et al. 2009, Schoebel et al. 2010, Del Campo et al. 2014) and the phytate-activated protein kinase Lpg2603 (Hubber et al. 2014, Sreelatha et al. 2020) bind to PtdIns(4)*P*. On the other hand, the retromer interactor RidL (Finsel et al. 2013), the Atg8 phosphatidylethanolamine deconjugase RavC (Choy et al. 2012, Horenkamp et al. 2015), and the glycosyltransferases SetA (Jank et al. 2012) and LtpM (Levanova et al. 2019) bind to PtdIns(3)*P*.

Upon maturation, the LCV undergoes a conversion from a tight to a spacious compartment (Lu and Clarke 2005, Ragaz et al. 2008, Case et al. 2016) and intimately associates with the ER (Swanson and Isberg 1995, Abu Kwaik 1996, Solomon and Isberg 2000, Lu and Clarke 2005, Robinson and Roy 2006). The ER does not fuse with the PtdIns(4)*P*-positive LCV membrane for at least 8 h post infection (Weber et al. 2014) (Fig. 4). Intriguingly, the contact sites of the LCV with the ER are connected by periodic ‘hair-like’ structures (Tilney et al. 2001), and ER elements remain attached to LCVs even after immuno-affinity purification of intact pathogen vacuoles (Urwyler et al. 2009, Hoffmann et al. 2014b, Schmölders et al. 2017). Taken together, these findings suggested that the LCV forms MCS with the ER.

Using a proteomics approach and dually fluorescence-labelled *D. discoideum* amoeba, Vormittag *et al*. recently analysed the role of selected MCS proteins for LCV-ER MCS formation and vacuole remodelling (Vormittag et al. 2023). Comparative proteomics analysis of LCVs purified from a *D. discoideum* parental strain or from a strain lacking the ER-residing large fusion GTPase Sey1/atlastin (Steiner et al. 2017, Hüsler et al. 2021) indicated the presence of the MCS proteins OSBP7, OSBP8 and the PtdIns(4)*P* 4-phosphatase Sac1 (Vormittag et al. 2023). The study also revealed that at LCV-ER MCS VAP localized to both the ER and the LCV membrane, while Sac1, OSBP7, and OSBP8 preferentially localized to the ER, and OSBP11 preferentially localized to the LCV membrane (Fig. 4).

VAP, Sac1 and OSBP11 promoted initial LCV expansion and intracellular replication of *L. pneumophila*, whereas OSBP8 restricted these processes (Vormittag et al. 2023). Furthermore, staining with the sterol probes filipin and GFP-D4H* (Tweten 1988, Shatursky et al. 1999) revealed that sterols are depleted from the LCV within 2 h post infection in the parental *D. discoideum* strain, as well as in mutant strains lacking VAP, OSBP7, OSBP8 or OSBP11, while PtdIns(4)*P* accumulated in parallel (Vormittag et al. 2023). In addition to Sac1, the *L. pneumophila* PtdIns 4-kinase LepB and the PtdIns(4)*P*-binding effector SidC also promoted initial LCV expansion, since *L. pneumophila* mutant strains lacking these effectors were impaired for this process (Fig. 4). In summary, the study indicated that a *Legionella*- and host cell-driven PtdIns(4)*P* gradient generated at LCV-ER MCS promotes VAP-, OSBP- and Sac1-dependent LCV maturation (Vormittag et al. 2023).

Finally, *L. pneumophila* effectors not only localize to LCV-ER MCS but also target mitochondria-ER contact sites (Murata et al. 2022). Mitochondria-ER-associated membranes (MAMs) are implicated in various cellular functions, including lipid synthesis and trafficking, mitochondrial morphology, inflammasome activation, autophagosome formation, and apoptosis (Escoll et al. 2017). The *L. pneumophila* effector Lpg1137 binds the MAM- and mitochondria-enriched phospholipid phosphatidic acid and proteolytically degrades the MAM-localizing SNARE syntaxin 17 (Murata et al. 2022).

### Membrane contact sites of the *Coxiella*-containing vacuole

*Coxiella burnetii* is a Gram-negative, coccobacillary, obligate intracellular bacterium and the causative agent of Q fever (Delsing et al. 2011). After uptake, *C. burnetii* resides within a replication-permissive compartment, the *Coxiella*-containing vacuole (CCV) (Heinzen et al. 1999, Voth and Heinzen 2007). The nascent CCV fuses with early and late endosomes, lysosomes and autophagosomes and adopts an acidic pH of ∼4.5, which activates the Icm/Dot T4BSS (Voth and Heinzen 2007, Newton et al. 2013) and the translocation of over 130 effector proteins into the host cytoplasm (Segal et al. 2005, Beare et al. 2011, Qiu and Luo 2017).

ORP1L is recruited to the CCV in a T4BSS-dependent manner prior to pathogen vacuole expansion (Justis et al. 2017). The association of ORP1L with the CCV occurs through its N-terminal ARD domain, which interacts with active Rab7 localized at the CCV (Beron et al. 2002, Johansson et al. 2005, Justis et al. 2017). Because ORP1L contains a FFAT motif and binds to VAP on the ER, it is possible that ORP1L is part of a protein complex that mediates lipid transfer and/or tethering of the CCV to the ER, leading to the formation of CCV-ER MCS.

The role of ORP1L in lipid transfer to the CCV is supported by the fact that the CCV is rich in sterols, as determined by filipin staining, and by the reduction of CCV size upon depletion of ORP1L, although bacterial growth was not affected (Justis et al. 2017). The reduction of *C. burnetii* growth observed upon knockdown of the cholesterol transporter NPC-1 or pharmacological depletion of cholesterol, does however support the importance of sterols for bacterial growth (Howe and Heinzen 2006, Czyz et al. 2014).

It is not known if *Coxiella* effectors plays a role in ORP1L recruitment to the CCV or in CCV-ER MCS biology at wide. Of the many *Coxiella* effectors only a few have been characterized (Qiu and Luo 2017). Of interest, the Icm/Dot substrate ElpA (ER-localizing protein A) is present in most *C. burnetii* strains and disrupts ER structure and function during infection (Graham et al. 2015). Additionally, the *C. burnetii* effector *Coxiella* vacuolar protein B (CvpB) binds PtdIns(3)*P* and PS on CCVs and early endosomal compartments (Martinez et al. 2016). CvpB also inhibits the activity of the PtdIns 5-kinase PIKfyve to manipulate PtdIns(3)*P* metabolism and to promote CCV expansion. Based on the preliminary characterization of ElpA and CvpB, it will be interesting to investigate their potential role in CCV-ER interactions.

### Modulation of the *Salmonella*-containing vacuole by OSBP and VAP

*Salmonella enterica* serovar Typhimurium (*S*. Typhimurium) is a Gram-negative, facultative intracellular bacterial pathogen and the cause of gastroenteritis and diarrhea (Hohmann 2001). During infection, *S*. Typhimurium forms a replication-permissive compartment, the *Salmonella*-containing vacuole (SCV) in phagocytic and epithelial cells (Knodler and Steele-Mortimer 2003, Bakowski et al. 2008, LaRock et al. 2015). SCV formation is controlled by two type III secretion systems (T3SSs), located on *Salmonella* pathogenicity island (SPI)1 and SPI2 (Jennings et al. 2017, Galan and Waksman 2018). SPI1 is required for the initial invasion, and SPI2 is produced after internalization. SPI2 translocates approximately 30 different effector proteins into the host cytoplasm (LaRock et al. 2015, Jennings et al. 2017).

Bacterial growth in the SCV is promoted by effectors translocated by the SPI2 T3SS. These effectors prevent fusion with bactericidal lysosomes, direct the SCV to close proximity with the Golgi apparatus and trigger the formation of membrane tubules (*Salmonella*-induced filaments, Sifs), a process, which requires the effectors SseF and SseG (Kuhle et al. 2004, Deiwick et al. 2006). SseF and SseG as well as SifA have been shown to intercept secretory trafficking from the Golgi to the PM (Kuhle et al. 2006, Bakowski et al. 2008). SifA, SseJ and to a lesser extend SseL are also necessary for cholesterol accumulation at the SCV (McEwan et al. 2015, Walch et al. 2021). SseJ esterifies cholesterol due to its deacylase activity and increases the formation of LDs (Ohlson et al. 2005, Nawabi et al. 2008). This enzymatic activity and the localization of SseJ to the SCV requires binding to active RhoA GTPase (LaRock et al. 2012).

OSBP localizes to the SCV in a process mediated by SseJ and SseL (Auweter et al. 2012, Kolodziejek et al. 2019). SseJ binds OSBP at the coiled-coil domain independent of RhoA (Kolodziejek et al. 2019), and SseL binds OSBP at the coiled-coil domain as well as at the FFAT motif (Rytkonen et al. 2007, Auweter et al. 2012). OSBP has been shown to support intracellular replication of *S*. Typhimurium (Auweter et al. 2012). Additionally, infection of OSBP-depleted, or VAPA/B double knockout cells resulted in increased cytoplasmic *S*. Typhimurium, suggesting a stabilization role of OSBP and VAPA/B for the SCV (Kolodziejek et al. 2019). While these findings suggest the formation of SCV-ER MCS, they could also reflect indirect effects via ER-Golgi MCS disruption, calling for future studies to further characterize SCV-ER interactions.

## Conclusions

MCS are characterized by discrete stretches of membrane contact between two apposing organelles to facilitate the non-vesicular trafficking of small molecules such as calcium and lipids. The extensive characterization of a multitude of MCS in mammalian cells and yeast that occurred over the past decade, resulted in a comprehensive, yet constantly evolving, structural, molecular, and functional landscape of the MCS. Lipid transfer at MCS is a complex and highly regulated process. By anchoring to each of the contacting organelles, via binding to receptors (e.g. VAP) on one organelle, and specific PI lipids on the other, specific lipid transfer proteins (e.g. OSBP, ORPs, CERT) mediate the transfer of lipids (e.g. sterols, PtdIns(4)*P*, PE, ceramide, etc) from one organelle to another. Phosphoinositide phosphatases such as Sac1 further modify the lipid composition of the donor or recipient membrane. The short range (10-30 nm) lipid exchange that establishes along a gradient is key to membrane remodelling and organelle maturation to adopt specific functions.

Intravacuolar bacterial pathogens such as *Chlamydia, Legionella, Coxiella*, and *Salmonella*, have evolved to mimic and/or hijack these non-vesicular trafficking processes to establish their distinct replication-permissive compartments. The mechanisms reported so far include the formation of MCS between the pathogen vacuole and the ER, the recruitment of specific cellular MCS components to pathogen vacuoles aided by the translocation of bacterial effector proteins, and/or the establishment of a lipid gradient between the pathogen vacuole and the ER.

Future studies will continue to investigate the complex composition and architecture of MCS in naïve or infected cells, using proteomics approaches as well as high-resolution fluorescence microscopy and cryo-EM technology. To assess functional aspects of MCS, bacterial effectors targeting MCS components might serve as versatile tools. Hence, in addition to generating cell biological insights into MCS components, architecture and function, the sophisticated ways bacterial pathogens subvert MCS will also be elucidated.

## References

[bib1] Abdelrahman YM , Belland RJ. The chlamydial developmental cycle. FEMS Microbiol Rev. 2005;29:949–59. 16043254 10.1016/j.femsre.2005.03.002

[bib2] Abu Kwaik Y . The phagosome containing *Legionella pneumophila* within the protozoan *Hartmannella vermiformis* is surrounded by the rough endoplasmic reticulum. Appl Environ Microbiol. 1996;62:2022–8. 8787400 10.1128/aem.62.6.2022-2028.1996 PMC167980

[bib3] Agaisse H , Derré I. Expression of the effector protein IncD in *Chlamydia trachomatis* mediates recruitment of the lipid transfer protein CERT and the endoplasmic reticulum-resident protein VAPB to the inclusion membrane. Infect Immun. 2014;82:2037–47. 24595143 10.1128/IAI.01530-14 PMC3993449

[bib4] Agaisse H , Derré I. STIM1 Is a novel component of ER-*Chlamydia trachomatis* inclusion membrane contact sites. PLoS One. 2015;10:e0125671. 25915399 10.1371/journal.pone.0125671 PMC4411163

[bib5] Amako Y , Sarkeshik A, Hotta H et al. Role of oxysterol binding protein in hepatitis C virus infection. J Virol. 2009;83:9237–46. 19570870 10.1128/JVI.00958-09 PMC2738263

[bib6] Area-Gomez E , Del Carmen Lara Castillo M, Tambini MD et al. Upregulated function of mitochondria-associated ER membranes in Alzheimer disease. EMBO J. 2012;31:4106–23. 22892566 10.1038/emboj.2012.202 PMC3492725

[bib7] Auweter SD , Yu HB, Arena ET et al. Oxysterol-binding protein (OSBP) enhances replication of intracellular *Salmonella* and binds the *Salmonella* SPI-2 effector SseL via its N-terminus. Microbes Infect. 2012;14:148–54. 21988961 10.1016/j.micinf.2011.09.003

[bib8] Bakowski MA , Braun V, Brumell JH. *Salmonella*-containing vacuoles: directing traffic and nesting to grow. Traffic. 2008;9:2022–31. 18778407 10.1111/j.1600-0854.2008.00827.x

[bib9] Balla T . Phosphoinositides: tiny lipids with giant impact on cell regulation. Physiol Rev. 2013;93:1019–137. 23899561 10.1152/physrev.00028.2012 PMC3962547

[bib10] Bannantine JP , Griffiths RS, Viratyosin W et al. A secondary structure motif predictive of protein localization to the chlamydial inclusion membrane. Cell Microbiol. 2000;2:35–47. 11207561 10.1046/j.1462-5822.2000.00029.x

[bib11] Bärlocher K , Welin A, Hilbi H. Formation of the *Legionella* replicative compartment at the crossroads of retrograde trafficking. Front. Cell. Infect. Microbiol. 2017;7:482. 29226112 10.3389/fcimb.2017.00482 PMC5706426

[bib12] Beare PA , Gilk SD, Larson CL et al. Dot/Icm type IVB secretion system requirements for *Coxiella burnetii* growth in human macrophages. MBio. 2011;2:e00175–11. 21862628 10.1128/mBio.00175-11 PMC3163939

[bib13] Behnia R , Munro S. Organelle identity and the signposts for membrane traffic. Nature. 2005;438:597–604. 16319879 10.1038/nature04397

[bib14] Bernhard W , Rouiller C. Close topographical relationship between mitochondria and ergastoplasm of liver cells in a definite phase of cellular activity. J Biophys Biochem Cytol. 1956;2:73–78. 13357525 10.1083/jcb.2.4.73 PMC2229714

[bib15] Beron W , Gutierrez MG, Rabinovitch M et al. *Coxiella burnetii* localizes in a Rab7-labeled compartment with autophagic characteristics. Infect Immun. 2002;70:5816–21. 12228312 10.1128/IAI.70.10.5816-5821.2002 PMC128334

[bib16] Birge RB , Boeltz S, Kumar S et al. Phosphatidylserine is a global immunosuppressive signal in efferocytosis, infectious disease, and cancer. Cell Death Differ. 2016;23:962–78. 26915293 10.1038/cdd.2016.11 PMC4987730

[bib17] Blagoveshchenskaya A , Cheong FY, Rohde HM et al. Integration of Golgi trafficking and growth factor signaling by the lipid phosphatase SAC1. J Cell Biol. 2008;180:803–12. 18299350 10.1083/jcb.200708109 PMC2265582

[bib18] Boamah DK , Zhou G, Ensminger AW et al. From many hosts, one accidental pathogen: the diverse protozoan hosts of *Legionella*. Front. Cell. Infect. Microbiol. 2017;7:477. 29250488 10.3389/fcimb.2017.00477 PMC5714891

[bib19] Bonifacino JS , Lippincott-Schwartz J. Coat proteins: shaping membrane transport. Nat Rev Mol Cell Biol. 2003;4:409–14. 12728274 10.1038/nrm1099

[bib20] Brombacher E , Urwyler S, Ragaz C et al. Rab1 guanine nucleotide exchange factor SidM is a major phosphatidylinositol 4-phosphate-binding effector protein of *Legionella pneumophila*. J Biol Chem. 2009;284:4846–56. 19095644 10.1074/jbc.M807505200 PMC2643517

[bib21] Brouckaert G , Kalai M, Krysko DV et al. Phagocytosis of necrotic cells by macrophages is phosphatidylserine dependent and does not induce inflammatory cytokine production. MBoC. 2004;15:1089–100. 14668480 10.1091/mbc.E03-09-0668 PMC363082

[bib22] Bugalhao JN , Mota LJ. The multiple functions of the numerous *Chlamydia trachomatis* secreted proteins: the tip of the iceberg. Microb Cell. 2019;6:414–49. 31528632 10.15698/mic2019.09.691 PMC6717882

[bib23] Cabukusta B , Berlin I, van Elsland DM et al. Human VAPome analysis reveals MOSPD1 and MOSPD3 as membrane contact site proteins interacting with FFAT-related FFNT motifs. Cell Rep. 2020;33:108475. 33296653 10.1016/j.celrep.2020.108475

[bib24] Case ED , Smith JA, Ficht TA et al. Space: a final frontier for vacuolar pathogens. Traffic. 2016;17:461–74. 26842840 10.1111/tra.12382 PMC6048968

[bib25] Castro IG , Richards DM, Metz J et al. A role for mitochondrial Rho GTPase 1 (MIRO1) in motility and membrane dynamics of peroxisomes. Traffic. 2018;19:229–42. 29364559 10.1111/tra.12549 PMC5888202

[bib26] Chamberlain NB , Dimond Z, Hackstadt T. *Chlamydia trachomatis* suppresses host cell store-operated Ca(2+) entry and inhibits NFAT/calcineurin signaling. Sci Rep. 2022;12:21406. 36496532 10.1038/s41598-022-25786-y PMC9741641

[bib27] Charman M , Colbourne TR, Pietrangelo A et al. Oxysterol-binding protein (OSBP)-related protein 4 (ORP4) is essential for cell proliferation and survival. J Biol Chem. 2014;289:15705–17. 24742681 10.1074/jbc.M114.571216 PMC4140924

[bib28] Cheong FY , Sharma V, Blagoveshchenskaya A et al. Spatial regulation of Golgi phosphatidylinositol-4-phosphate is required for enzyme localization and glycosylation fidelity. Traffic. 2010;11:1180–90. 20573065 10.1111/j.1600-0854.2010.01092.x PMC2921771

[bib29] Cheong HC , Lee CYQ, Cheok YY et al. Chlamydiaceae: diseases in primary hosts and zoonosis. Microorganisms. 2019;7:146. 31137741 10.3390/microorganisms7050146 PMC6560403

[bib30] Choi WY , Kim S, Aurass P et al. SdhA blocks disruption of the *Legionella*-containing vacuole by hijacking the OCRL phosphatase. Cell Rep. 2021;37:109894. 34731604 10.1016/j.celrep.2021.109894 PMC8669613

[bib31] Choy A , Dancourt J, Mugo B et al. The *Legionella* effector RavZ inhibits host autophagy through irreversible Atg8 deconjugation. Science. 2012;338:1072–6. 23112293 10.1126/science.1227026 PMC3682818

[bib32] Christoforidis S , Miaczynska M, Ashman K et al. Phosphatidylinositol-3-OH kinases are Rab5 effectors. Nat Cell Biol. 1999;1:249–52. 10559924 10.1038/12075

[bib33] Chung J , Torta F, Masai K et al. Intracellular transport. PI4P/phosphatidylserine countertransport at ORP5- and ORP8-mediated ER-plasma membrane contacts. Science. 2015;349:428–32. 26206935 10.1126/science.aab1370 PMC4638224

[bib34] Copeland D E , Dalton AJ. An association between mitochondria and the endoplasmic reticulum in cells of the pseudobranch gland of a teleost. J Biophys Biochem Cytol. 1959;5:393–6. 13664679 10.1083/jcb.5.3.393 PMC2224680

[bib35] Cortina ME , Derré I. Homologues of the *Chlamydia trachomatis* and *Chlamydia muridarum* inclusion membrane protein IncS are interchangeable for early development but not for inclusion stability in the late developmental cycle. mSphere. 2023:e0000323. 36853051 10.1128/msphere.00003-23 PMC10117133

[bib36] Czyz DM , Potluri LP, Jain-Gupta N et al. Host-directed antimicrobial drugs with broad-spectrum efficacy against intracellular bacterial pathogens. MBio. 2014;5:e01534–14. 25073644 10.1128/mBio.01534-14 PMC4128363

[bib37] D'Souza RS , Lim JY, Turgut A et al. Calcium-stimulated disassembly of focal adhesions mediated by an ORP3/IQSec1 complex. Elife. 2020;9:e54113. 32234213 10.7554/eLife.54113 PMC7159923

[bib38] D'Ambrosio JM , Albanèse V, Lipp NF et al. Osh6 requires Ist2 for localization to ER–PM contacts and efficient phosphatidylserine transport in budding yeast. J Cell Sci. 2020;133:jcs243733. 32327560 10.1242/jcs.243733

[bib39] De Matteis MA , Godi A. PI-loting membrane traffic. Nat Cell Biol. 2004;6:487–92. 15170460 10.1038/ncb0604-487

[bib40] de Saint-Jean M , Delfosse V, Douguet D et al. Osh4p exchanges sterols for phosphatidylinositol 4-phosphate between lipid bilayers. J Cell Biol. 2011;195:965–78. 22162133 10.1083/jcb.201104062 PMC3241724

[bib41] Dehoux P , Flores R, Dauga C et al. Multi-genome identification and characterization of chlamydiae-specific type III secretion substrates: the Inc proteins. Bmc Genomics [Electronic Resource]. 2011;12:109. 21324157 10.1186/1471-2164-12-109 PMC3048545

[bib42] Deiwick J , Salcedo SP, Boucrot E et al. The translocated *Salmonella* effector proteins SseF and SseG interact and are required to establish an intracellular replication niche. Infect Immun. 2006;74:6965–72. 17015457 10.1128/IAI.00648-06 PMC1698088

[bib43] Del Campo CM , Mishra AK, Wang YH et al. Structural basis for PI(4)P-specific membrane recruitment of the *Legionella pneumophila* effector DrrA/SidM. Structure. 2014;22:397–408. 24530282 10.1016/j.str.2013.12.018 PMC3974903

[bib44] Delsing CE , Warris A, Bleeker-Rovers CP. Q fever: still more queries than answers. Adv Exp Med Biol. 2011;719:133–43. 22125041 10.1007/978-1-4614-0204-6_12

[bib45] Derré I . Hijacking of membrane contact sites by intracellular bacterial pathogens. Adv Exp Med Biol. 2017;997:211–23. 28815533 10.1007/978-981-10-4567-7_16

[bib46] Derré I , Swiss R, Agaisse H. The lipid transfer protein CERT interacts with the *Chlamydia* inclusion protein IncD and participates to ER-*Chlamydia* inclusion membrane contact sites. PLoS Pathog. 2011;7:e1002092. 21731489 10.1371/journal.ppat.1002092 PMC3121800

[bib47] Di Mattia T , Wilhelm LP, Ikhlef S et al. Identification of MOSPD2, a novel scaffold for endoplasmic reticulum membrane contact sites. EMBO Rep. 2018;19:e45453. 29858488 10.15252/embr.201745453 PMC6030701

[bib48] Di Mattia T , Martinet A, Ikhlef S et al. FFAT motif phosphorylation controls formation and lipid transfer function of inter-organelle contacts. EMBO J. 2020;39:e104369. 33124732 10.15252/embj.2019104369 PMC7705450

[bib49] Di Paolo G , De Camilli P. Phosphoinositides in cell regulation and membrane dynamics. Nature. 2006;443:651–7. 17035995 10.1038/nature05185

[bib50] Dolinsky S , Haneburger I, Cichy A et al. The *Legionella longbeachae* Icm/Dot substrate SidC selectively binds phosphatidylinositol 4-phosphate with nanomolar affinity and promotes pathogen vacuole-endoplasmic reticulum interactions. Infect Immun. 2014;82:4021–33. 25024371 10.1128/IAI.01685-14 PMC4187854

[bib51] Dong J , Du X, Wang H et al. Allosteric enhancement of ORP1-mediated cholesterol transport by PI(4,5)P2/PI(3,4)P2. Nat Commun. 2019;10:829. 30783101 10.1038/s41467-019-08791-0 PMC6381110

[bib52] Dong N , Niu M, Hu L et al. Modulation of membrane phosphoinositide dynamics by the phosphatidylinositide 4-kinase activity of the *Legionella* LepB effector. Nat Microbiol. 2016a;2:16236. 27941800 10.1038/nmicrobiol.2016.236

[bib53] Dong R , Saheki Y, Swarup S et al. Endosome-ER contacts control actin nucleation and retromer function through VAP-dependent regulation of PI4P. Cell. 2016;166:408–23. 27419871 10.1016/j.cell.2016.06.037 PMC4963242

[bib54] Du X , Kumar J, Ferguson C et al. A role for oxysterol-binding protein-related protein 5 in endosomal cholesterol trafficking. J Cell Biol. 2011;192:121–35. 21220512 10.1083/jcb.201004142 PMC3019559

[bib55] Du X , Zhou L, Aw YC et al. ORP5 localizes to ER-lipid droplet contacts and regulates the level of PI(4)P on lipid droplets. J Cell Biol. 2020;219:e201905162. 31653673 10.1083/jcb.201905162 PMC7039201

[bib56] Duan G , Walther D. The roles of post-translational modifications in the context of protein interaction networks. PLoS Comput Biol. 2015;11:e1004049. 25692714 10.1371/journal.pcbi.1004049 PMC4333291

[bib57] Dumoux M , Clare DK, Saibil HR et al. *Chlamydiae* assemble a pathogen synapse to hijack the host endoplasmic reticulum. Traffic. 2012;13:1612–27. 22901061 10.1111/tra.12002 PMC3533787

[bib58] Dumoux M , Nans A, Saibil HR et al. Making connections: snapshots of chlamydial type III secretion systems in contact with host membranes. Curr Opin Microbiol. 2015;23:1–7. 25461566 10.1016/j.mib.2014.09.019

[bib59] Eden ER , Sanchez-Heras E, Tsapara A et al. Annexin A1 tethers membrane contact sites that mediate ER to endosome cholesterol transport. Dev Cell. 2016;37:473–83. 27270042 10.1016/j.devcel.2016.05.005 PMC4906250

[bib60] Eisenberg-Bord M , Shai N, Schuldiner M et al. A tether is a tether is a tether: tethering at membrane contact sites. Dev Cell. 2016;39:395–409. 27875684 10.1016/j.devcel.2016.10.022

[bib61] Elwell CA , Engel JN. Lipid acquisition by intracellular *Chlamydiae*. Cell Microbiol. 2012;14:1010–8. 22452394 10.1111/j.1462-5822.2012.01794.x PMC3376245

[bib62] Elwell CA , Jiang S, Kim JH et al. *Chlamydia trachomatis* co-opts GBF1 and CERT to acquire host sphingomyelin for distinct roles during intracellular development. PLoS Pathog. 2011;7:e1002198. 21909260 10.1371/journal.ppat.1002198 PMC3164637

[bib63] Ende RJ , Murray RL, D'Spain SK. Phosphoregulation accommodates type III secretion and assembly of a tether of ER-*Chlamydia* inclusion membrane contact sites. Elife. 2022;11:e74535. 35838228 10.7554/eLife.74535 PMC9286742

[bib64] Escoll P , Mondino S, Rolando M et al. Targeting of host organelles by pathogenic bacteria: a sophisticated subversion strategy. Nat Rev Microbiol. 2016;14:5–19. 26594043 10.1038/nrmicro.2015.1

[bib65] Escoll P , Rolando M, Buchrieser C. MAMs are attractive targets for bacterial repurposing of the host cell: MAM-functions might be key for undermining an infected cell. Bioessays. 2017;39:1600171 doi: 10.1002/bies.201600171. 10.1002/bies.201600171 28026026

[bib66] Finsel I , Hilbi H. Formation of a pathogen vacuole according to *Legionella pneumophila*: how to kill one bird with many stones. Cell Microbiol. 2015;17:935–50. 25903720 10.1111/cmi.12450

[bib67] Finsel I , Ragaz C, Hoffmann C et al. The *Legionella* effector RidL inhibits retrograde trafficking to promote intracellular replication. Cell Host Microbe. 2013;14:38–50. 23870312 10.1016/j.chom.2013.06.001

[bib68] Friedman JR , Webster BM, Mastronarde DN et al. ER sliding dynamics and ER-mitochondrial contacts occur on acetylated microtubules. J Cell Biol. 2010;190:363–75. 20696706 10.1083/jcb.200911024 PMC2922647

[bib69] Friedman JR , Lackner LL, West M et al. ER tubules mark sites of mitochondrial division. Science. 2011;334:358–62. 21885730 10.1126/science.1207385 PMC3366560

[bib70] Fukuzawa M , Williams JG. OSBPa, a predicted oxysterol binding protein of *Dictyostelium*, is required for regulated entry into culmination. FEBS Lett. 2002;527:37–42. 12220630 10.1016/s0014-5793(02)03150-2

[bib71] Funato K , Riezman H, Muniz M. Vesicular and non-vesicular lipid export from the ER to the secretory pathway. Biochimica et Biophysica Acta (BBA) - Mol Cell Biol Lip. 2020;1865:158453. 10.1016/j.bbalip.2019.04.013 31054928

[bib72] Galan JE , Waksman G. Protein-injection machines in bacteria. Cell. 2018;172:1306–18. 29522749 10.1016/j.cell.2018.01.034 PMC5849082

[bib73] Galmes R , Houcine A, van Vliet AR et al. ORP5/ORP8 localize to endoplasmic reticulum-mitochondria contacts and are involved in mitochondrial function. EMBO Rep. 2016;17:800–10. 27113756 10.15252/embr.201541108 PMC5278607

[bib74] Ge J , Shao F. Manipulation of host vesicular trafficking and innate immune defence by *Legionella* Dot/Icm effectors. Cell Microbiol. 2011;13:1870–80. 21981078 10.1111/j.1462-5822.2011.01710.x

[bib75] Ghai R , Du X, Wang H et al. ORP5 and ORP8 bind phosphatidylinositol-4,5-biphosphate (PtdIns(4,5)P 2) and regulate its level at the plasma membrane. Nat Commun. 2017;8:757. 28970484 10.1038/s41467-017-00861-5 PMC5624964

[bib76] Giles DK , Wyrick PB. Trafficking of chlamydial antigens to the endoplasmic reticulum of infected epithelial cells. Microbes Infect. 2008;10:1494–503. 18832043 10.1016/j.micinf.2008.09.001 PMC2645044

[bib77] Giorgi C , Bonora M, Sorrentino G et al. p53 at the endoplasmic reticulum regulates apoptosis in a Ca2+-dependent manner. Proc Natl Acad Sci USA. 2015;112:1779–84. 25624484 10.1073/pnas.1410723112 PMC4330769

[bib78] Gitsels A , Sanders N, Vanrompay D. Chlamydial infection from outside to inside. Front Microbiol. 2019;10:2329. 31649655 10.3389/fmicb.2019.02329 PMC6795091

[bib79] Godi A , Pertile P, Meyers R et al. ARF mediates recruitment of PtdIns-4-OH kinase-beta and stimulates synthesis of PtdIns(4,5)P2 on the Golgi complex. Nat Cell Biol. 1999;1:280–7. 10559940 10.1038/12993

[bib80] Goto A , Liu X, Robinson CA et al. Multisite phosphorylation of oxysterol-binding protein regulates sterol binding and activation of sphingomyelin synthesis. MBoC. 2012;23:3624–35. 22875984 10.1091/mbc.E12-04-0283 PMC3442410

[bib81] Graham JG , Winchell CG, Sharma UM et al. Identification of ElpA, a *Coxiella burnetii* pathotype-specific Dot/Icm type IV secretion system substrate. Infect Immun. 2015;83:1190–8. 25605765 10.1128/IAI.02855-14 PMC4333474

[bib82] Gulyas G , Sohn M, Kim YJ et al. ORP3 phosphorylation regulates phosphatidylinositol 4-phosphate and Ca(2+) dynamics at plasma membrane-ER contact sites. J Cell Sci. 2020;133:jcs237388. 32041906 10.1242/jcs.237388 PMC7097422

[bib83] Hammer JA 3rd , Wu XS. Rabs grab motors: defining the connections between Rab GTPases and motor proteins. Curr Opin Cell Biol. 2002;14:69–75. 11792547 10.1016/s0955-0674(01)00296-4

[bib84] Hanada K . Lipid transfer proteins rectify inter-organelle flux and accurately deliver lipids at membrane contact sites. J Lipid Res. 2018;59:1341–66. 29884707 10.1194/jlr.R085324 PMC6071762

[bib85] Hanada K , Kumagai K, Yasuda S et al. Molecular machinery for non-vesicular trafficking of ceramide. Nature. 2003;426:803–9. 14685229 10.1038/nature02188

[bib86] Heinzen RA , Hackstadt T, Samuel JE. Developmental biology of *Coxiella burnettii*. Trends Microbiol. 1999;7:149–54. 10217829 10.1016/s0966-842x(99)01475-4

[bib87] Hilbi H , Haas A. Secretive bacterial pathogens and the secretory pathway. Traffic. 2012;13:1187–97. 22340894 10.1111/j.1600-0854.2012.01344.x

[bib88] Hilbi H , Buchrieser C. Microbe profile: *Legionella pneumophila* - a copycat eukaryote. Microbiology. 2022;168: doi: 10.1099/mic.0.001142 10.1099/mic.0.001142 35230931

[bib89] Hilbi H , Hoffmann C, Harrison CF. *Legionella* spp. outdoors: colonization, communication and persistence. Environ Microbiol Rep. 2011;3:286–96. 23761274 10.1111/j.1758-2229.2011.00247.x

[bib90] Hoffmann C , Harrison CF, Hilbi H. The natural alternative: protozoa as cellular models for *Legionella* infection. Cell Microbiol. 2014;16:15–26. 24168696 10.1111/cmi.12235

[bib91] Hoffmann C , Finsel I, Otto A et al. Functional analysis of novel Rab GTPases identified in the proteome of purified *Legionella*-containing vacuoles from macrophages. Cell Microbiol. 2014b;16:1034–52s. 24373249 10.1111/cmi.12256

[bib92] Hohmann EL . Nontyphoidal salmonellosis. Clin Infect Dis. 2001;32:263–9. 11170916 10.1086/318457

[bib93] Holthuis JC , Menon AK. Lipid landscapes and pipelines in membrane homeostasis. Nature. 2014;510:48–57. 24899304 10.1038/nature13474

[bib94] Honscher C , Ungermann C. A close-up view of membrane contact sites between the endoplasmic reticulum and the endolysosomal system: from yeast to man. Crit Rev Biochem Mol Biol. 2014;49:262–8. 24382115 10.3109/10409238.2013.875512

[bib95] Honscher C , Mari M, Auffarth K et al. Cellular metabolism regulates contact sites between vacuoles and mitochondria. Dev Cell. 2014;30:86–94. 25026035 10.1016/j.devcel.2014.06.006

[bib96] Horenkamp FA , Kauffman KJ, Kohler LJ et al. The *Legionella* anti-autophagy effector RavZ targets the autophagosome via PI3P- and curvature-sensing motifs. Dev Cell. 2015;34:569–76. 26343456 10.1016/j.devcel.2015.08.010 PMC4594837

[bib97] Howe D , Heinzen RA. *Coxiella burnetii* inhabits a cholesterol-rich vacuole and influences cellular cholesterol metabolism. Cell Microbiol. 2006;8:496–507. 16469060 10.1111/j.1462-5822.2005.00641.x

[bib98] Hsu F , Zhu W, Brennan L et al. Structural basis for substrate recognition by a unique *Legionella* phosphoinositide phosphatase. Proc Natl Acad Sci USA. 2012;109:13567–72. 22872863 10.1073/pnas.1207903109 PMC3427105

[bib99] Hubber A , Roy CR. Modulation of host cell function by *Legionella pneumophila* type IV effectors. Annu Rev Cell Dev Biol. 2010;26:261–83. 20929312 10.1146/annurev-cellbio-100109-104034

[bib100] Hubber A , Arasaki K, Nakatsu F et al. The machinery at endoplasmic reticulum-plasma membrane contact sites contributes to spatial regulation of multiple *Legionella* effector proteins. PLoS Pathog. 2014;10:e1004222. 24992562 10.1371/journal.ppat.1004222 PMC4081824

[bib101] Hüsler D , Steiner B, Welin A et al. *Dictyostelium* lacking the single atlastin homolog Sey1 shows aberrant ER architecture, proteolytic processes and expansion of the *Legionella*-containing vacuole. Cell Microbiol. 2021;23:e13318. 33583106 10.1111/cmi.13318

[bib102] Hynynen R , Suchanek M, Spandl J et al. OSBP-related protein 2 is a sterol receptor on lipid droplets that regulates the metabolism of neutral lipids. J Lipid Res. 2009;50:1305–15. 19224871 10.1194/jlr.M800661-JLR200 PMC2694330

[bib103] Hynynen R , Laitinen S, Kakela R et al. Overexpression of OSBP-related protein 2 (ORP2) induces changes in cellular cholesterol metabolism and enhances endocytosis. Biochem J. 2005;390:273–83. 15859942 10.1042/BJ20042082 PMC1184581

[bib104] Isberg RR , O'Connor TJ, Heidtman M. The *Legionella pneumophila* replication vacuole: making a cosy niche inside host cells. Nat Rev Microbiol. 2009;7:13–24. 19011659 10.1038/nrmicro1967 PMC2631402

[bib105] Ishikawa-Sasaki K , Nagashima S, Taniguchi K et al. Model of OSBP-mediated cholesterol supply to Aichi virus RNA replication sites involving protein-protein interactions among viral proteins, ACBD3, OSBP, VAP-A/B, and SAC1. J Virol. 2018;92:e01952–17. 29367253 10.1128/JVI.01952-17 PMC5874406

[bib106] Jahn R , Scheller RH. SNAREs–engines for membrane fusion. Nat Rev Mol Cell Biol. 2006;7:631–43. 16912714 10.1038/nrm2002

[bib107] James C , Kehlenbach RH. The interactome of the VAP family of proteins: an overview. Cells. 2021;10:1780. 34359948 10.3390/cells10071780 PMC8306308

[bib108] Jank T , Bohmer KE, Tzivelekidis T et al. Domain organization of *Legionella* effector SetA. Cell Microbiol. 2012;14:852–68. 22288428 10.1111/j.1462-5822.2012.01761.x

[bib109] Jansen M , Ohsaki Y, Rega LR et al. Role of ORPs in sterol transport from plasma membrane to ER and lipid droplets in mammalian cells. Traffic. 2011;12:218–31. 21062391 10.1111/j.1600-0854.2010.01142.x

[bib110] Jaworski CJ , Moreira E, Li A et al. A family of 12 human genes containing oxysterol-binding domains. Genomics. 2001;78:185–96. 11735225 10.1006/geno.2001.6663

[bib111] Jeckel D , Karrenbauer A, Birk R et al. Sphingomyelin is synthesized in the cis Golgi. FEBS Lett. 1990;261:155–7. 2155131 10.1016/0014-5793(90)80659-7

[bib112] Jennings E , Thurston TLM, Holden DW. *Salmonella* SPI-2 type III secretion system effectors: molecular mechanisms and physiological consequences. Cell Host Microbe. 2017;22:217–31. 28799907 10.1016/j.chom.2017.07.009

[bib113] Johansson M , Lehto M, Tanhuanpaa K et al. The oxysterol-binding protein homologue ORP1L interacts with Rab7 and alters functional properties of late endocytic compartments. MBoC. 2005;16:5480–92. 16176980 10.1091/mbc.E05-03-0189 PMC1289395

[bib114] Johansson M , Bocher V, Lehto M et al. The two variants of oxysterol binding protein-related protein-1 display different tissue expression patterns, have different intracellular localization, and are functionally distinct. MBoC. 2003;14:903–15. 12631712 10.1091/mbc.E02-08-0459 PMC151568

[bib115] Jones DH , Morris JB, Morgan CP et al. Type I phosphatidylinositol 4-phosphate 5-kinase directly interacts with ADP-ribosylation factor 1 and is responsible for phosphatidylinositol 4,5-bisphosphate synthesis in the Golgi compartment. J Biol Chem. 2000;275:13962–6. 10747863 10.1074/jbc.c901019199

[bib116] Justis AV , Hansen B, Beare PA et al. Interactions between the *Coxiella burnetii* parasitophorous vacuole and the endoplasmic reticulum involve the host protein ORP1L. Cell Microbiol. 2017;19:e12637 10.1111/cmi.12637. 10.1111/cmi.12637 PMC5177503 27345457

[bib117] Kaiser SE , Brickner JH, Reilein AR et al. Structural basis of FFAT motif-mediated ER targeting. Structure. 2005;13:1035–45. 16004875 10.1016/j.str.2005.04.010

[bib118] Kawano M , Kumagai K, Nishijima M et al. Efficient trafficking of ceramide from the endoplasmic reticulum to the Golgi apparatus requires a VAMP-associated protein-interacting FFAT motif of CERT. J Biol Chem. 2006;281:30279–88. 16895911 10.1074/jbc.M605032200

[bib119] Kirchhausen T . Three ways to make a vesicle. Nat Rev Mol Cell Biol. 2000;1:187–98. 11252894 10.1038/35043117

[bib120] Knodler LA , Steele-Mortimer O. Taking possession: biogenesis of the *Salmonella*-containing vacuole. Traffic. 2003;4:587–99. 12911813 10.1034/j.1600-0854.2003.00118.x

[bib121] Kolodziejek AM , Altura MA, Fan J et al. *Salmonella* translocated effectors recruit OSBP1 to the phagosome to promote vacuolar membrane integrity. Cell Rep. 2019;27:2147–2156.e5. 31091452 10.1016/j.celrep.2019.04.021

[bib122] Konrad G , Schlecker T, Faulhammer F et al. Retention of the yeast Sac1p phosphatase in the endoplasmic reticulum causes distinct changes in cellular phosphoinositide levels and stimulates microsomal ATP transport. J Biol Chem. 2002;277:10547–54. 11792713 10.1074/jbc.M200090200

[bib123] Kors S , Hacker C, Bolton C et al. Regulating peroxisome-ER contacts via the ACBD5-VAPB tether by FFAT motif phosphorylation and GSK3beta. J Cell Biol. 2022;221:e202003143. 35019937 10.1083/jcb.202003143 PMC8759595

[bib124] Kreutzberger AJ , Kiessling V, Tamm LK. High cholesterol obviates a prolonged hemifusion intermediate in fast SNARE-mediated membrane fusion. Biophys J. 2015;109:319–29. 26200867 10.1016/j.bpj.2015.06.022 PMC4621810

[bib125] Kubori T , Nagai H. The type IVB secretion system: an enigmatic chimera. Curr Opin Microbiol. 2016;29:22–29. 26529574 10.1016/j.mib.2015.10.001

[bib126] Kudo N , Kumagai K, Tomishige N et al. Structural basis for specific lipid recognition by CERT responsible for nonvesicular trafficking of ceramide. Proc Natl Acad Sci USA. 2008;105:488–93. 18184806 10.1073/pnas.0709191105 PMC2206563

[bib127] Kuhle V , Jackel D, Hensel M. Effector proteins encoded by *Salmonella* pathogenicity island 2 interfere with the microtubule cytoskeleton after translocation into host cells. Traffic. 2004;5:356–70. 15086785 10.1111/j.1398-9219.2004.00179.x

[bib128] Kuhle V , Abrahams GL, Hensel M. Intracellular *Salmonella enterica* redirect exocytic transport processes in a *Salmonella* pathogenicity island 2-dependent manner. Traffic. 2006;7:716–30. 16637890 10.1111/j.1600-0854.2006.00422.x

[bib129] Kumagai K , Kawano-Kawada M, Hanada K. Phosphoregulation of the ceramide transport protein CERT at serine 315 in the interaction with VAMP-associated protein (VAP) for inter-organelle trafficking of ceramide in mammalian cells. J Biol Chem. 2014;289:10748–60. 24569996 10.1074/jbc.M113.528380 PMC4036191

[bib130] Laitinen S , Olkkonen VM, Ehnholm C et al. Family of human oxysterol binding protein (OSBP) homologues: a novel member implicated in brain sterol metabolism. J Lipid Res. 1999;40:2204–11. 10588946

[bib131] Laitinen S , Lehto M, Lehtonen S et al. ORP2, a homolog of oxysterol binding protein, regulates cellular cholesterol metabolism. J Lipid Res. 2002;43:245–55. 11861666

[bib132] Lara-Tejero M , Galan JE. The injectisome, a complex nanomachine for protein injection into mammalian cells. EcoSal Plus. 2019;8:10.1128. PMID: 30942149. 10.1128/ecosalplus.esp-0039-2018 PMC6450406 30942149

[bib133] LaRock DL , Chaudhary A, Miller SI. *Salmonellae* interactions with host processes. Nat Rev Microbiol. 2015;13:191–205. 25749450 10.1038/nrmicro3420 PMC5074537

[bib134] LaRock DL , Brzovic PS, Levin I et al. A *Salmonella typhimurium*-translocated glycerophospholipid:cholesterol acyltransferase promotes virulence by binding to the RhoA protein switch regions. J Biol Chem. 2012;287:29654–63. 22740689 10.1074/jbc.M112.363598 PMC3436183

[bib135] Lee M , Fairn GD. Both the PH domain and N-terminal region of oxysterol-binding protein related protein 8S are required for localization to PM-ER contact sites. Biochem Biophys Res Commun. 2018;496:1088–94. 29409900 10.1016/j.bbrc.2018.01.138

[bib136] Lehto M , Tienari J, Lehtonen S et al. Subfamily III of mammalian oxysterol-binding protein (OSBP) homologues: the expression and intracellular localization of ORP3, ORP6, and ORP7. Cell Tissue Res. 2004;315:39–57. 14593528 10.1007/s00441-003-0817-y

[bib137] Lehto M , Hynynen R, Karjalainen K et al. Targeting of OSBP-related protein 3 (ORP3) to endoplasmic reticulum and plasma membrane is controlled by multiple determinants. Exp Cell Res. 2005;310:445–62. 16143324 10.1016/j.yexcr.2005.08.003

[bib138] Lehto M , Laitinen S, Chinetti G et al. The OSBP-related protein family in humans. J Lipid Res. 2001;42:1203–13. 11483621

[bib139] Lehto M , Mayranpaa MI, Pellinen T et al. The R-Ras interaction partner ORP3 regulates cell adhesion. J Cell Sci. 2008;121:695–705. 18270267 10.1242/jcs.016964

[bib140] Lemmon MA . Pleckstrin homology domains: not just for phosphoinositides. Biochem Soc Trans. 2004;32:707–11. 15493994 10.1042/BST0320707

[bib141] Levanova N , Mattheis C, Carson D et al. The *Legionella* effector LtpM is a new type of phosphoinositide-activated glucosyltransferase. J Biol Chem. 2019;294:2862–5740. 30573678 10.1074/jbc.RA118.005952 PMC6393602

[bib142] Leventis PA , Grinstein S. The distribution and function of phosphatidylserine in cellular membranes. Annu Rev Biophys. 2010;39:407–27. 20192774 10.1146/annurev.biophys.093008.131234

[bib143] Lewis SC , Uchiyama LF, Nunnari J. ER-mitochondria contacts couple mtDNA synthesis with mitochondrial division in human cells. Science. 2016;353:aaf5549. 27418514 10.1126/science.aaf5549 PMC5554545

[bib144] Li G , Liu H, Luo ZQ et al. Modulation of phagosome phosphoinositide dynamics by a *Legionella* phosphoinositide 3-kinase. EMBO Rep. 2021;22:e51163. 33492731 10.15252/embr.202051163 PMC7926237

[bib145] Liu X , Ridgway ND. Characterization of the sterol and phosphatidylinositol 4-phosphate binding properties of Golgi-associated OSBP-related protein 9 (ORP9). PLoS One. 2014;9:e108368. 25255026 10.1371/journal.pone.0108368 PMC4177916

[bib146] Loewen CJ , Levine TP. A highly conserved binding site in vesicle-associated membrane protein-associated protein (VAP) for the FFAT motif of lipid-binding proteins. J Biol Chem. 2005;280:14097–104. 15668246 10.1074/jbc.M500147200

[bib147] Loewen CJ , Roy A, Levine TP. A conserved ER targeting motif in three families of lipid binding proteins and in Opi1p binds VAP. EMBO J. 2003;22:2025–35. 12727870 10.1093/emboj/cdg201 PMC156073

[bib148] Lu H , Clarke M. Dynamic properties of *Legionella*-containing phagosomes in *Dictyostelium* amoebae. Cell Microbiol. 2005;7:995–1007. 15953031 10.1111/j.1462-5822.2005.00528.x

[bib149] Luo X , Wasilko DJ, Liu Y et al. Structure of the *Legionella* virulence factor, SidC reveals a unique PI(4)P-specific binding domain essential for its targeting to the bacterial phagosome. PLoS Pathog. 2015;11:e1004965. 26067986 10.1371/journal.ppat.1004965 PMC4467491

[bib150] Lutter EI , Martens C, Hackstadt T. Evolution and conservation of predicted inclusion membrane proteins in *chlamydiae*. Comp Funct Genomics. 2012;2012:1. 10.1155/2012/362104 PMC3290821 22454599

[bib151] Ma W , Mayr C. A membraneless organelle associated with the endoplasmic reticulum enables 3'UTR-mediated protein-protein interactions. Cell. 2018;175:1492–1506.e19. 30449617 10.1016/j.cell.2018.10.007 PMC6711188

[bib152] Maeda K , Anand K, Chiapparino A et al. Interactome map uncovers phosphatidylserine transport by oxysterol-binding proteins. Nature. 2013;501:257–61. 23934110 10.1038/nature12430

[bib153] Malhotra M , Sood S, Mukherjee A et al. Genital *Chlamydia trachomatis*: an update. Indian J Med Res. 2013;138:303–16. 24135174 PMC3818592

[bib154] Martinez E , Allombert J, Cantet F et al. *Coxiella burnetii* effector CvpB modulates phosphoinositide metabolism for optimal vacuole development. Proc Natl Acad Sci USA. 2016;113:E3260–3269. 27226300 10.1073/pnas.1522811113 PMC4988616

[bib155] Matanis T , Akhmanova A, Wulf P et al. Bicaudal-D regulates COPI-independent Golgi-ER transport by recruiting the dynein-dynactin motor complex. Nat Cell Biol. 2002;4:986–92. 12447383 10.1038/ncb891

[bib156] McCune BT , Tang W, Lu J et al. Noroviruses co-opt the function of host proteins VAPA and VAPB for replication via a phenylalanine-phenylalanine-acidic-tract-motif mimic in nonstructural viral protein NS1/2. MBio. 2017;8:e00668–17. 28698274 10.1128/mBio.00668-17 PMC5513711

[bib157] McEwan DG , Richter B, Claudi B et al. PLEKHM1 regulates *Salmonella*-containing vacuole biogenesis and infection. Cell Host Microbe. 2015;17:58–71. 25500191 10.1016/j.chom.2014.11.011

[bib158] McMahon HT , Mills IG. COP and clathrin-coated vesicle budding: different pathways, common approaches. Curr Opin Cell Biol. 2004;16:379–91. 15261670 10.1016/j.ceb.2004.06.009

[bib159] Mesmin B , Maxfield FR. Intracellular sterol dynamics. Biochimica et Biophysica Acta (BBA) - Mol Cell Biol Lip. 2009;1791:636–45. 10.1016/j.bbalip.2009.03.002 PMC2696574 19286471

[bib160] Mesmin B , Bigay J, Moser von Filseck J et al. A four-step cycle driven by PI(4)P hydrolysis directs sterol/PI(4)P exchange by the ER-Golgi tether OSBP. Cell. 2013;155:830–43. 24209621 10.1016/j.cell.2013.09.056

[bib161] Mesmin B , Bigay J, Polidori J et al. Sterol transfer, PI4P consumption, and control of membrane lipid order by endogenous OSBP. EMBO J. 2017;36:3156–74. 28978670 10.15252/embj.201796687 PMC5666618

[bib162] Mirrashidi KM , Elwell CA, Verschueren E et al. Global mapping of the Inc-human interactome reveals that retromer restricts *Chlamydia* infection. Cell Host Microbe. 2015;18:109–21. 26118995 10.1016/j.chom.2015.06.004 PMC4540348

[bib163] Mishori R , McClaskey EL, WinklerPrins VJ. *Chlamydia trachomatis* infections: screening, diagnosis, and management. Am Fam Physician. 2012;86:1127–32. 23316985

[bib164] Mondino S , Schmidt S, Rolando M et al. Legionnaires' disease: state of the art knowledge of pathogenesis mechanisms of *Legionella*. Annu Rev Pathol Mech Dis. 2020;15:439–66. 10.1146/annurev-pathmechdis-012419-032742 31657966

[bib165] Moore ER , Ouellette SP. Reconceptualizing the chlamydial inclusion as a pathogen-specified parasitic organelle: an expanded role for Inc proteins. Front Cell Infect Microbiol. 2014;4:157. 25401095 10.3389/fcimb.2014.00157 PMC4215707

[bib166] Moser von Filseck J , Copic A, Delfosse V et al. Intracellular transport. Phosphatidylserine transport by ORP/Osh proteins is driven by phosphatidylinositol 4-phosphate. Science. 2015;349:432–6. 26206936 10.1126/science.aab1346

[bib167] Moulder JW . Interaction of *Chlamydiae* and host cells in vitro. Microbiol Rev. 1991;55:143–90. 2030670 10.1128/mr.55.1.143-190.1991 PMC372804

[bib168] Murata M , Kanamori R, Kitao T et al. Requirement of phosphatidic acid binding for distribution of the bacterial protein Lpg1137 targeting syntaxin 17. J Cell Sci. 2022;135:jcs259538. 35224642 10.1242/jcs.259538

[bib169] Murley A , Lackner LL, Osman C et al. ER-associated mitochondrial division links the distribution of mitochondria and mitochondrial DNA in yeast. Elife. 2013;2:e00422. 23682313 10.7554/eLife.00422 PMC3654481

[bib170] Murphy SE , Levine TP. VAP, a versatile access point for the endoplasmic reticulum: review and analysis of FFAT-like motifs in the VAPome. Biochimica et Biophysica Acta (BBA) - Mol Cell Biol Lip. 2016;1861:952–61. 10.1016/j.bbalip.2016.02.009 26898182

[bib171] Murray JT , Panaretou C, Stenmark H et al. Role of Rab5 in the recruitment of hVps34/p150 to the early endosome. Traffic. 2002;3:416–27. 12010460 10.1034/j.1600-0854.2002.30605.x

[bib172] Nawabi P , Catron DM, Haldar K. Esterification of cholesterol by a type III secretion effector during intracellular *Salmonella* infection. Mol Microbiol. 2008;68:173–85. 18333886 10.1111/j.1365-2958.2008.06142.x

[bib173] Nemoto Y , Kearns BG, Wenk MR et al. Functional characterization of a mammalian Sac1 and mutants exhibiting substrate-specific defects in phosphoinositide phosphatase activity. J Biol Chem. 2000;275:34293–305. 10887188 10.1074/jbc.M003923200

[bib174] Newton HJ , McDonough JA, Roy CR. Effector protein translocation by the *Coxiella burnetii* Dot/Icm type IV secretion system requires endocytic maturation of the pathogen-occupied vacuole. PLoS One. 2013;8:e54566. 23349930 10.1371/journal.pone.0054566 PMC3547880

[bib175] Newton HJ , Ang DK, van Driel IR et al. Molecular pathogenesis of infections caused by *Legionella pneumophila*. Clin Microbiol Rev. 2010;23:274–98. 20375353 10.1128/CMR.00052-09 PMC2863363

[bib176] Ngo M , Ridgway ND. Oxysterol binding protein-related protein 9 (ORP9) is a cholesterol transfer protein that regulates Golgi structure and function. MBoC. 2009;20:1388–99. 19129476 10.1091/mbc.E08-09-0905 PMC2649274

[bib177] Nguyen PH , Lutter EI, Hackstadt T. *Chlamydia trachomatis* inclusion membrane protein MrcA interacts with the inositol 1,4,5-trisphosphate receptor type 3 (ITPR3) to regulate extrusion formation. PLoS Pathog. 2018;14:e1006911. 29543918 10.1371/journal.ppat.1006911 PMC5854415

[bib178] Nissila E , Ohsaki Y, Weber-Boyvat M et al. ORP10, a cholesterol binding protein associated with microtubules, regulates apolipoprotein B-100 secretion. Biochimica et Biophysica Acta (BBA) - Mol Cell Biol Lip. 2012;1821:1472–84. 10.1016/j.bbalip.2012.08.004 22906437

[bib179] Ohlson MB , Fluhr K, Birmingham CL et al. SseJ deacylase activity by *Salmonella enterica* serovar Typhimurium promotes virulence in mice. Infect Immun. 2005;73:6249–59. 16177296 10.1128/IAI.73.10.6249-6259.2005 PMC1230951

[bib180] Olkkonen VM , Li S. Oxysterol-binding proteins: sterol and phosphoinositide sensors coordinating transport, signaling and metabolism. Prog Lipid Res. 2013;52:529–38. 23830809 10.1016/j.plipres.2013.06.004

[bib181] Olkkonen VM , Beaslas O, Nissila E. Oxysterols and their cellular effectors. Biomolecules. 2012;2:76–103. 24970128 10.3390/biom2010076 PMC4030866

[bib182] Personnic N , Bärlocher K, Finsel I et al. Subversion of retrograde trafficking by translocated pathogen effectors. Trends Microbiol. 2016;24:450–62. 26924068 10.1016/j.tim.2016.02.003

[bib183] Peterson EM , de la Maza LM. *Chlamydia* parasitism: ultrastructural characterization of the interaction between the chlamydial cell envelope and the host cell. J Bacteriol. 1988;170:1389–92. 3343223 10.1128/jb.170.3.1389-1392.1988 PMC210922

[bib184] Phillips MJ , Voeltz GK. Structure and function of ER membrane contact sites with other organelles. Nat Rev Mol Cell Biol. 2016;17:69–82. 26627931 10.1038/nrm.2015.8 PMC5117888

[bib185] Pietrangelo A , Ridgway ND. Golgi localization of oxysterol binding protein-related protein 4 L (ORP4L) is regulated by ligand binding. J Cell Sci. 2018;131:jcs215335. 29930082 10.1242/jcs.215335

[bib186] Pietrangelo A , Ridgway ND. Phosphorylation of a serine/proline-rich motif in oxysterol binding protein-related protein 4 L (ORP4L) regulates cholesterol and vimentin binding. PLoS One. 2019;14:e0214768. 30925160 10.1371/journal.pone.0214768 PMC6440634

[bib187] Prakriya M . The theory, operation, and roles of store-operated calcium. Curr Top Membr. 2013;71:xi–xii. 23890119 10.1016/B978-0-12-407870-3.10000-9

[bib188] Prinz WA . Bridging the gap: membrane contact sites in signaling, metabolism, and organelle dynamics. J Cell Biol. 2014;205:759–69. 24958771 10.1083/jcb.201401126 PMC4068136

[bib189] Prinz WA , Toulmay A, Balla T. The functional universe of membrane contact sites. Nat Rev Mol Cell Biol. 2020;21:7–24. 31732717 10.1038/s41580-019-0180-9 PMC10619483

[bib190] Qiu J , Luo ZQ. *Legionella* and *Coxiella* effectors: strength in diversity and activity. Nat Rev Microbiol. 2017;15:591–605. 28713154 10.1038/nrmicro.2017.67

[bib191] Radhakrishnan A , Goldstein JL, McDonald JG et al. Switch-like control of SREBP-2 transport triggered by small changes in ER cholesterol: a delicate balance. Cell Metab. 2008;8:512–21. 19041766 10.1016/j.cmet.2008.10.008 PMC2652870

[bib192] Ragaz C , Pietsch H, Urwyler S et al. The *Legionella pneumophila* phosphatidylinositol-4 phosphate-binding type IV substrate SidC recruits endoplasmic reticulum vesicles to a replication-permissive vacuole. Cell Microbiol. 2008;10:2416–33. 18673369 10.1111/j.1462-5822.2008.01219.x

[bib193] Raiborg C , Wenzel EM, Pedersen NM et al. Phosphoinositides in membrane contact sites. Biochem Soc Trans. 2016;44:425–30. 27068950 10.1042/BST20150190

[bib194] Rizzuto R , Pinton P, Carrington W et al. Close contacts with the endoplasmic reticulum as determinants of mitochondrial Ca2+ responses. Science. 1998;280:1763–6. 9624056 10.1126/science.280.5370.1763

[bib195] Robinson CG , Roy CR. Attachment and fusion of endoplasmic reticulum with vacuoles containing *Legionella pneumophila*. Cell Microbiol. 2006;8:793–805. 16611228 10.1111/j.1462-5822.2005.00666.x

[bib196] Rocha N , Kuijl C, van der Kant R et al. Cholesterol sensor ORP1L contacts the ER protein VAP to control Rab7-RILP-p150 glued and late endosome positioning. J Cell Biol. 2009;185:1209–25. 19564404 10.1083/jcb.200811005 PMC2712958

[bib197] Rohde HM , Cheong FY, Konrad G et al. The human phosphatidylinositol phosphatase SAC1 interacts with the coatomer I complex. J Biol Chem. 2003;278:52689–99. 14527956 10.1074/jbc.M307983200

[bib198] Roulin PS , Lötzerich M, Torta F et al. Rhinovirus uses a phosphatidylinositol 4-phosphate/cholesterol counter-current for the formation of replication compartments at the ER-Golgi interface. Cell Host & Microbe. 2014;16:677–90. 25525797 10.1016/j.chom.2014.10.003

[bib199] Rowland AA , Chitwood PJ, Phillips MJ et al. ER contact sites define the position and timing of endosome fission. Cell. 2014;159:1027–41. 25416943 10.1016/j.cell.2014.10.023 PMC4634643

[bib200] Rytkonen A , Poh J, Garmendia J et al. SseL, a *Salmonella* deubiquitinase required for macrophage killing and virulence. Proc Natl Acad Sci USA. 2007;104:3502–7. 17360673 10.1073/pnas.0610095104 PMC1802004

[bib201] Schmölders J , Manske C, Otto A et al. Comparative proteomics of purified pathogen vacuoles correlates intracellular replication of *Legionella pneumophila* with the small GTPase Ras-related protein 1 (Rap1). Mol Cell Proteomics. 2017;16:622–41. 28183814 10.1074/mcp.M116.063453 PMC5383783

[bib202] Schoebel S , Blankenfeldt W, Goody RS et al. High-affinity binding of phosphatidylinositol 4-phosphate by *Legionella pneumophila* DrrA. EMBO Rep. 2010;11:598–604. 20616805 10.1038/embor.2010.97 PMC2920447

[bib203] Scorrano L , De Matteis MA, Emr S et al. Coming together to define membrane contact sites. Nat Commun. 2019;10:1287. 30894536 10.1038/s41467-019-09253-3 PMC6427007

[bib204] Segal G , Feldman M, Zusman T. The Icm/Dot type-IV secretion systems of *Legionella pneumophila* and *Coxiella burnetii*. FEMS Microbiol Rev. 2005;29:65–81. 15652976 10.1016/j.femsre.2004.07.001

[bib205] Shai N , Yifrach E, van Roermund CWT et al. Systematic mapping of contact sites reveals tethers and a function for the peroxisome-mitochondria contact. Nat Commun. 2018;9:1761. 29720625 10.1038/s41467-018-03957-8 PMC5932058

[bib206] Shatursky O , Heuck AP, Shepard LA et al. The mechanism of membrane insertion for a cholesterol-dependent cytolysin: a novel paradigm for pore-forming toxins. Cell. 1999;99:293–9. 10555145 10.1016/s0092-8674(00)81660-8

[bib207] Short B , Preisinger C, Schaletzky J et al. The Rab6 GTPase regulates recruitment of the dynactin complex to Golgi membranes. Curr Biol. 2002;12:1792–5. 12401177 10.1016/s0960-9822(02)01221-6

[bib208] Slee JA , Levine TP. Systematic prediction of FFAT motifs across eukaryote proteomes identifies nucleolar and eisosome proteins with the predicted capacity to form bridges to the endoplasmic reticulum. Contact. 2019;2:1–21. 31777772 10.1177/2515256419883136 PMC6881177

[bib209] Sohn M , Korzeniowski M, Zewe JP et al. PI(4,5)P2 controls plasma membrane PI4P and PS levels via ORP5/8 recruitment to ER-PM contact sites. J Cell Biol. 2018;217:1797–813. 29472386 10.1083/jcb.201710095 PMC5940310

[bib210] Solomon JM , Isberg RR. Growth of *Legionella pneumophila* in *Dictyostelium discoideum*: a novel system for genetic analysis of host-pathogen interactions. Trends Microbiol. 2000;8:478–80. 11044684 10.1016/s0966-842x(00)01852-7

[bib211] Springer S , Spang A, Schekman R. A primer on vesicle budding. Cell. 1999;97:145–8. 10219233 10.1016/s0092-8674(00)80722-9

[bib212] Sreelatha A , Nolan C, Park BC et al. A *Legionella* effector kinase is activated by host inositol hexakisphosphate. J Biol Chem. 2020;295:6214–24. 32229585 10.1074/jbc.RA120.013067 PMC7196655

[bib213] Srikanth S , Gwack Y. Orai1, STIM1, and their associating partners. J Physiol. 2012;590:4169–77. 22586216 10.1113/jphysiol.2012.231522 PMC3473276

[bib214] Stanhope R , Derré I. Making contact: VAP targeting by intracellular pathogens. Contact. 2018;1:251525641877551. 10.1177/2515256418775512 PMC6083021 30101212

[bib215] Stanhope R , Flora E, Bayne C et al. IncV, a FFAT motif-containing *Chlamydia* protein, tethers the endoplasmic reticulum to the pathogen-containing vacuole. Proc Natl Acad Sci USA. 2017;114:12039–44. 29078338 10.1073/pnas.1709060114 PMC5692559

[bib216] Steiner B , Weber S, Hilbi H. Formation of the *Legionella*-containing vacuole: phosphoinositide conversion, GTPase modulation and ER dynamics. Int J Med Microbiol. 2018;308:49–57. 28865995 10.1016/j.ijmm.2017.08.004

[bib217] Steiner B , Swart AL, Welin A et al. ER remodeling by the large GTPase atlastin promotes vacuolar growth of *Legionella pneumophila*. EMBO Rep. 2017;18:1817–36. 28835546 10.15252/embr.201743903 PMC5623866

[bib218] Steinert M , Heuner K. *Dictyostelium* as host model for pathogenesis. Cell Microbiol. 2005;7:307–14. 15679834 10.1111/j.1462-5822.2005.00493.x

[bib219] Stoica R , De Vos KJ, Paillusson S et al. ER-mitochondria associations are regulated by the VAPB-PTPIP51 interaction and are disrupted by ALS/FTD-associated TDP-43. Nat Commun. 2014;5:3996. 24893131 10.1038/ncomms4996 PMC4046113

[bib220] Stratton BS , Warner JM, Wu Z et al. Cholesterol increases the openness of SNARE-mediated flickering fusion pores. Biophys J. 2016;110:1538–50. 27074679 10.1016/j.bpj.2016.02.019 PMC4833774

[bib221] Striednig B , Lanner U, Niggli S et al. Quorum sensing governs a transmissive *Legionella* subpopulation at the pathogen vacuole periphery. EMBO Rep. 2021; 22:e52972. 34314090 10.15252/embr.202152972 PMC8419707

[bib222] Suchanek M , Hynynen R, Wohlfahrt G et al. The mammalian oxysterol-binding protein-related proteins (ORPs) bind 25-hydroxycholesterol in an evolutionarily conserved pocket. Biochem J. 2007;405:473–80. 17428193 10.1042/BJ20070176 PMC2267293

[bib223] Swanson MS , Isberg RR. Association of *Legionella pneumophila* with the macrophage endoplasmic reticulum. Infect Immun. 1995;63:3609–20. 7642298 10.1128/iai.63.9.3609-3620.1995 PMC173501

[bib224] Swart AL , Hilbi H. Phosphoinositides and the fate of *Legionella* in phagocytes. Front Immunol. 2020;11:25. 32117224 10.3389/fimmu.2020.00025 PMC7025538

[bib225] Swart AL , Gomez-Valero L, Buchrieser C et al. Evolution and function of bacterial RCC1 repeat effectors. Cell Microbiol. 2020;22:e13246. 32720355 10.1111/cmi.13246

[bib226] Swart AL , Harrison CF, Eichinger L et al. *Acanthamoeba* and *Dictyostelium* as cellular models for *Legionella* infection. Front Cell Infect Microbiol. 2018;8:61. 29552544 10.3389/fcimb.2018.00061 PMC5840211

[bib227] Taylor FR , Kandutsch AA. Oxysterol binding protein. Chem Phys Lipids. 1985;38:187–94. 4064220 10.1016/0009-3084(85)90066-0

[bib228] Tilney LG , Harb OS, Connelly PS et al. How the parasitic bacterium *Legionella pneumophila* modifies its phagosome and transforms it into rough ER: implications for conversion of plasma membrane to the ER membrane. J Cell Sci. 2001;114:4637–50. 11792828 10.1242/jcs.114.24.4637

[bib229] Tong J , Yang H, Yang H et al. Structure of Osh3 reveals a conserved mode of phosphoinositide binding in oxysterol-binding proteins. Structure. 2013;21:1203–13. 23791945 10.1016/j.str.2013.05.007

[bib230] Tweten RK . Cloning and expression in *Escherichia coli* of the perfringolysin O (theta-toxin) gene from *Clostridium perfringens* and characterization of the gene product. Infect Immun. 1988;56:3228–34. 2903127 10.1128/iai.56.12.3228-3234.1988 PMC259729

[bib231] Urwyler S , Nyfeler Y, Ragaz C et al. Proteome analysis of *Legionella* vacuoles purified by magnetic immunoseparation reveals secretory and endosomal GTPases. Traffic. 2009;10:76–87. 18980612 10.1111/j.1600-0854.2008.00851.x

[bib232] Valm AM , Cohen S, Legant WR et al. Applying systems-level spectral imaging and analysis to reveal the organelle interactome. Nature. 2017;546:162–7. 28538724 10.1038/nature22369 PMC5536967

[bib233] van Meer G , Voelker DR, Feigenson GW. Membrane lipids: where they are and how they behave. Nat Rev Mol Cell Biol. 2008;9:112–24. 18216768 10.1038/nrm2330 PMC2642958

[bib234] Vance JE . Phospholipid synthesis in a membrane fraction associated with mitochondria. J Biol Chem. 1990;265:7248–56. 2332429

[bib235] Venditti R , Rega LR, Masone MC et al. Molecular determinants of ER-Golgi contacts identified through a new FRET-FLIM system. J Cell Biol. 2019;218:1055–65. 30659100 10.1083/jcb.201812020 PMC6400564

[bib236] Vihervaara T , Uronen RL, Wohlfahrt G et al. Sterol binding by OSBP-related protein 1 L regulates late endosome motility and function. Cell Mol Life Sci. 2011;68:537–51. 20690035 10.1007/s00018-010-0470-z PMC11114714

[bib237] Vormittag S , Hüsler D, Haneburger I et al. *Legionella*- and host-driven lipid flux at LCV-ER membrane contact sites promotes vacuole remodeling. EMBO Rep. 2023;24:e56007. 36588479 10.15252/embr.202256007 PMC9986823

[bib238] Voth D E , Heinzen RA. Lounging in a lysosome: the intracellular lifestyle of *Coxiella burnetii*. Cell Microbiol. 2007;9:829–40. 17381428 10.1111/j.1462-5822.2007.00901.x

[bib239] Walch P , Selkrig J, Knodler LA et al. Global mapping of *Salmonella enterica*-host protein-protein interactions during infection. Cell Host Microbe. 2021;29:1316–1332.e12. 34237247 10.1016/j.chom.2021.06.004 PMC8561747

[bib240] Wang C , JeBailey L, Ridgway ND. Oxysterol-binding-protein (OSBP)-related protein 4 binds 25-hydroxycholesterol and interacts with vimentin intermediate filaments. Biochem J. 2002;361:461–72. 11802775 10.1042/0264-6021:3610461 PMC1222328

[bib241] Wang H , Ma Q, Qi Y et al. ORP2 delivers cholesterol to the plasma membrane in exchange for phosphatidylinositol 4, 5-bisphosphate (PI(4,5)P2). Mol Cell. 2019;73:458–473.e7. 30581148 10.1016/j.molcel.2018.11.014

[bib242] Wang M , Yang L, Chen Z et al. Geniposide ameliorates chronic unpredictable mild stress induced depression-like behavior through inhibition of ceramide-PP2A signaling via the PI3K/Akt/GSK3beta axis. Psychopharmacology (Berl). 2021;238:2789–800. 34142167 10.1007/s00213-021-05895-8

[bib243] Weber-Boyvat M , Kentala H, Lilja J et al. OSBP-related protein 3 (ORP3) coupling with VAMP-associated protein A regulates R-Ras activity. Exp Cell Res. 2015;331:278–91. 25447204 10.1016/j.yexcr.2014.10.019

[bib244] Weber S , Wagner M, Hilbi H. Live-cell imaging of phosphoinositide dynamics and membrane architecture during *Legionella* infection. MBio. 2014;5:e00839–13. 24473127 10.1128/mBio.00839-13 PMC3903275

[bib245] Weber SS , Ragaz C, Hilbi H. The inositol polyphosphate 5-phosphatase OCRL1 restricts intracellular growth of *Legionella*, localizes to the replicative vacuole and binds to the bacterial effector LpnE. Cell Microbiol. 2009;11:442–60. 19021631 10.1111/j.1462-5822.2008.01266.x

[bib246] Weber SS , Ragaz C, Hilbi H. Pathogen trafficking pathways and host phosphoinositide metabolism. Mol Microbiol. 2009;71:1341–52. 19208094 10.1111/j.1365-2958.2009.06608.x

[bib247] Weber SS , Ragaz C, Reus K et al. *Legionella pneumophila* exploits PI(4)P to anchor secreted effector proteins to the replicative vacuole. PLoS Pathog. 2006;2:e46. 16710455 10.1371/journal.ppat.0020046 PMC1463015

[bib248] Weixel KM , Blumental-Perry A, Watkins SC et al. Distinct Golgi populations of phosphatidylinositol 4-phosphate regulated by phosphatidylinositol 4-kinases. J Biol Chem. 2005;280:10501–8. 15634669 10.1074/jbc.M414304200

[bib249] Whitters EA , Cleves AE, McGee TP et al. SAC1p is an integral membrane protein that influences the cellular requirement for phospholipid transfer protein function and inositol in yeast. J Cell Biol. 1993;122:79–94. 8314848 10.1083/jcb.122.1.79 PMC2119615

[bib250] Wright HR , Turner A, Taylor HR. Trachoma. Lancet North Am Ed. 2008;371:1945–54. 10.1016/S0140-6736(08)60836-3 18539226

[bib251] Wu H , Carvalho P, Voeltz GK. Here, there, and everywhere: The importance of ER membrane contact sites. Science. 2018;361:eaan5835. 30072511 10.1126/science.aan5835 PMC6568312

[bib252] Wyles JP , Ridgway ND. VAMP-associated protein-A regulates partitioning of oxysterol-binding protein-related protein-9 between the endoplasmic reticulum and Golgi apparatus. Exp Cell Res. 2004;297:533–47. 15212954 10.1016/j.yexcr.2004.03.052

[bib253] Wyles JP , McMaster CR, Ridgway ND. Vesicle-associated membrane protein-associated protein-A (VAP-A) interacts with the oxysterol-binding protein to modify export from the endoplasmic reticulum. J Biol Chem. 2002;277:29908–18. 12023275 10.1074/jbc.M201191200

[bib254] Wyles JP , Perry RJ, Ridgway ND. Characterization of the sterol-binding domain of oxysterol-binding protein (OSBP)-related protein 4 reveals a novel role in vimentin organization. Exp Cell Res. 2007;313:1426–37. 17350617 10.1016/j.yexcr.2007.01.018

[bib255] Yan D , Mayranpaa MI, Wong J et al. OSBP-related protein 8 (ORP8) suppresses ABCA1 expression and cholesterol efflux from macrophages. J Biol Chem. 2008;283:332–40. 17991739 10.1074/jbc.M705313200

[bib256] Zhao K , Ridgway ND. Oxysterol-binding protein-related protein 1 L regulates cholesterol egress from the endo-lysosomal system. Cell Rep. 2017;19:1807–18. 28564600 10.1016/j.celrep.2017.05.028

[bib257] Zhao K , Foster J, Ridgway ND. Oxysterol-binding protein-related protein 1 variants have opposing cholesterol transport activities from the endolysosomes. MBoC. 2020;31:793–802. 32023146 10.1091/mbc.E19-12-0697 PMC7185962

[bib258] Zhong W , Yi Q, Xu B et al. ORP4L is essential for T-cell acute lymphoblastic leukemia cell survival. Nat Commun. 2016a;7:12702. 27581363 10.1038/ncomms12702 PMC5025801

[bib259] Zhong W , Pan G, Wang L et al. ORP4L facilitates macrophage survival via G-protein-coupled signaling: ORP4L-/- mice display a reduction of atherosclerosis. Circ Res. 2016;119:1296–312. 27729467 10.1161/CIRCRESAHA.116.309603

